# Nickel(ii)-catalyzed tandem C(sp^2^)–H bond activation and annulation of arenes with *gem*-dibromoalkenes†

**DOI:** 10.1039/c8ra03278e

**Published:** 2018-08-13

**Authors:** Yun Shi, Meng-Sheng Li, Fangdong Zhang, Baohua Chen

**Affiliations:** State Key Laboratory of Applied Organic Chemistry, Lanzhou University, Key Laboratory of Nonferrous Metal Chemistry and Resources Utilization of Gansu Province Lanzhou 730000 P. R. China chbh@lzu.edu.cn

## Abstract

A nickel(ii)/silver(i)-catalyzed tandem C(sp^2^)–H activation and intramolecular annulation of arenes with dibromoalkenes has been successfully achieved, which offers an efficient approach to the 3-methyleneisoindolin-1-one scaffold. Attractive features of this system include its low cost, ease of operation, and its ability to access a wide range of isoindolinones.

## Abstract 2

A nickel(ii)/silver(i)-catalyzed tandem C(sp^2^)–H activation and intramolecular annulation of arenes with dibromoalkenes has been successfully achieved, which offers an efficient approach to the 3-methyleneisoindolin-1-one scaffold.

## Graphical abstract

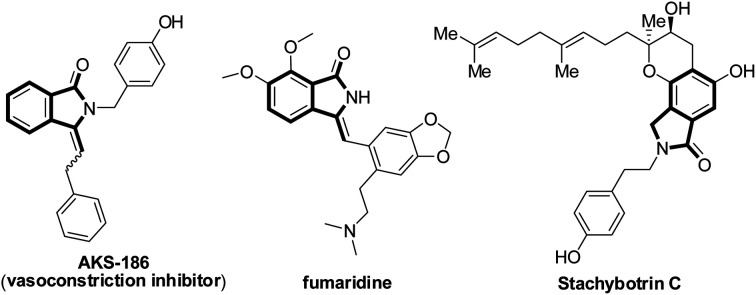

Over the past years, the transition-metal-catalyzed oxidative C–H/C–H cross-coupling reaction has emerged as a useful, atom- and step-economic synthetic protocol to construct a series of important N-heterocycles.^1^ In this context, the synthesis of isoindolinones has attracted considerable attention owing to their interesting biological and pharmaceutical properties,^2^ as well as their usefulness as precursors for the synthesis of structurally diverse and complex molecules (Scheme 1).^2*c*,3^ Several methods have successfully been developed toward isoindolinone synthesis based on Pd,^4^ Cu,^5^ Ru,^6^ and Rh^7^ salts. Among these reactions, the oxidative coupling reactions of benzamides with alkenes^4*b*,6,7*a*,*f*,*g*^ or alkynes^5*a*,5*d*^ exhibit high atom economy and the application of this strategy to simple arenes is still largely underdeveloped.^8^ For instance, in 2015, Zhang's group^9^ revealed cobalt-catalyzed oxidative alkynylation and cyclization of simple arenes and terminal alkynes with silver-cocatalyst *via* 2-fold C–H bond and N–H bond cleavage and C–C bond and C–N bond formation. In 2016, Song's group^10^ developed a method of a cobalt(ii)-catalyzed decarboxylative C–H activation/annulation of benzamides and alkynyl carboxylic acids and nickel(ii)-catalyzed C(sp^2^)–H alkynylation/annulation cascade with terminal alkynes to synthesize 3-methyleneiso-indolin-1-ones. Zhang also reported a nickel-catalyzed oxidative alkynylation with amides and terminal acetylenes.^10*c*^ In addition, from an environmentally point of view, in 2015, wei's group^11^ described an operationally simple, Pd-catalyzed C–H functionalization for the synthesis of important and useful isoindolinones from readily available carboxamides and carboxylic acids or anhydrides. The protocol avoided the use of excess oxidants including benzoquinone, Cu(OAc)_2_, or Ag_2_CO_3_ of previous all the reactions, thus generating stoichiometric amounts of undesired wastes.

**Scheme 1:**
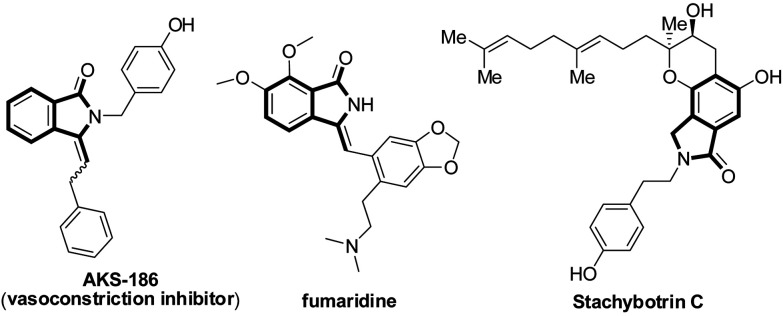
Representative isoindolinones with biological and pharmaceutical.

To our knowledge, the synthesis of alkynes is among the most fundamental and important synthetic transformations due to the unique reactivity of alkynes including addition, oxidation, reduction, and in particular cyclization.^12^ However, the lack of reactivity of alkynes, more electron-deficient than the corresponding alkenes, makes it harder to couple them with heteroarenes. As a consequence, terminal alkyne precursors have been developed to facilitate acetylene exchange.^13^ Halogenoalkynes,^14^ hypervalent alkynyliodoniums,^15^acetylenic sulfones,^16^ copper acetylides^17^ and α,β-ynoic acids^18^ allowed the generation of more activated alkyne moieties thus broadening the applications of direct alkynylation reactions to heterocycles. Among these alternatives, *gem*-dihaloalkenes emerged as more efficient coupling partners than the corresponding monohalogenated alkynes along with being inexpensive and readily-available.^19^ Indeed, the two geminal halogen atoms on the alkenyl carbon enhance the reactivity of metal complexes thus facilitating cross coupling reactions.^20^ Stable and readily-available 1,1-dibromo-1-alkenes and our interests in the C–H activation^21^ led us to consider using these reagents in the C–H functionalization to construct the valuable isoindolinones. We can envision that the abundance and structural diversity of the aldehydes (used the preparation of *gem*-dibromoethylenes *via* wittig reaction) as well as the merits of C–H functionalization would make the synthetic methods desirable and attractive. Herein, we wish to disclose the nickel(ii)/silver(i)-mediated tandem transformation involving sequential C(sp^2^)–H/C(sp^2^)–H alkynylation and intramolecular annulation of unactivated arenes with dibromoethylenes with the assistance of 8-aminoquinoline (Scheme 2). These features of this approach operational simplicity, a wide-ranging substrate scope, and tolerance of various synthetically useful functional groups.

**Scheme 2:**
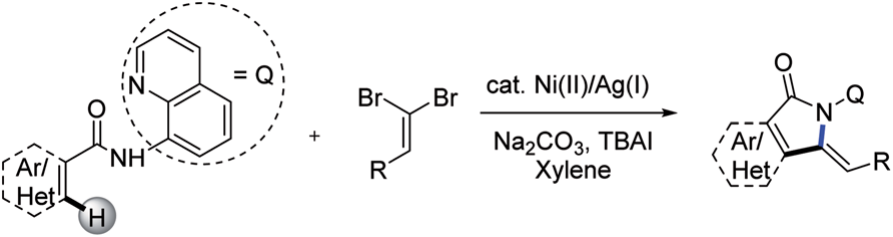
Nickel(ii)/silver(i)-catalyzed alkynylation/annulation of arenes with dibromoalkenes.

## Results and discussion

Initially, our investigation to explore the Ni(ii)-catalyzed C sp^2^–H activation began with the reaction of *N*-(quinolin-8-yl) benzamide (1a) and (2,2-dibromovinyl) benzene (2a) in the presence of selected catalysts as shown in Table 1. Gratifyingly, (*Z*)-3-benzylidene-2-(quinolin-8-yl) isoindolin-1-one 3a was obtained in 54% yield when NiCl·6H_2_O (10 mol%) was employed in xylene at 140 °C for 12 h (Table 1, entry 1). In order to search out an appropriate condition, we firstly screened a series of Ni(ii) salts and Ni(PPh_3_)_2_Cl_2_ (10 mol%) was chosen as the superior catalyst to give the corresponding product with a yield of 69% (entry 4). Moreover, the replacement of Ni(ii) salts with Cu(OAc)_2_ and Co(OAc)_2_·4H_2_O gave a slightly less yield (entries 5 and 6). These results reconfirmed that Ni(PPh_3_)_2_Cl_2_ is the optimal nickel catalyst source with a great advantage that other metal catalysts cannot match. Subsequently, the solvent effect was also evaluated and the promotional effect of the common solvents, such as DMF, DMSO, dioxane, and MeCN decreased drastically except chlorobenzene delivered 3a in up to 68% yield (entries 8–11). We suppose that the enhanced reactivity with aromatic solvent arises in part from its coordination with nickel catalyst, which would facilitate the formation of nickel chelate, intermediate. To further improve the conversion, we turned our attention to the oxidant effect and here we screened a number of silver(i) salts and another oxidant Cu(OAc)_2_, which showed Ag_2_CO_3_ was the best choice which promoted the formation of ring-closing reaction to give the desired product (entries 12–16). Next, we investigated the remarkable effect of additives using sodium iodide and different ammonium salts, however the reaction even did not work except TBAB with 58% yield, which implicated that the main role of TBAI in this transformation may be the phase transfer catalyst (entries 17–20). And finally, we examined the reaction under argon and oxygen atmosphere, obtained 37% and 41% yield respectively, which indicated the influence of gas atmosphere for the reaction was very low (entries 22 and 23). In addition, no reaction was observed in the absence of the nickel catalyst or silver salt and additive, indicating the necessity of both catalyst, Ag(i) salts and additives for the reaction (entries 7, 15, 21).

**Table 1:** Optimization of the reaction condition^a^

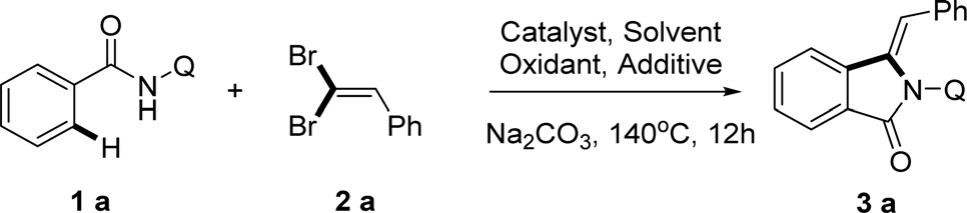					
Entry	Catalyst	Solvent	Oxidant	Additive	Yield (%)^b^
1	NiCl·6H_2_O	Xylene	Ag_2_CO_3_	TBAI	54
2	Ni(acac)_2_	Xylene	Ag_2_CO_3_	TBAI	62
3	Ni(OTf)_2_	Xylene	Ag_2_CO_3_	TBAI	52
**4**	**Ni(PPh**_**3**_ **)**_**2**_ **Cl**_**2**_	**Xylene**	**Ag**_**2**_ **CO**_**3**_	**TBAI**	**73**
5	Cu(OAc)_2_	Xylene	Ag_2_CO_3_	TBAI	42
6	Co(OAc)_2_·4H_2_O	Xylene	Ag_2_CO_3_	TBAI	31
7	—	Xylene	Ag_2_CO_3_	TBAI	0
8	Ni(PPh_3_)_2_Cl_2_	DMF	Ag_2_CO_3_	TBAI	Trace
9	Ni(PPh_3_)_2_Cl_2_	DMSO	Ag_2_CO_3_	TBAI	41
10	Ni(PPh_3_)_2_Cl_2_	PhCl	Ag_2_CO_3_	TBAI	68
11	Ni(PPh_3_)_2_Cl_2_	Dioxane	Ag_2_CO_3_	TBAI	56
12	Ni(PPh_3_)_2_Cl_2_	Xylene	AgOAc	TBAI	11
13	Ni(PPh_3_)_2_Cl_2_	Xylene	Ag_2_O	TBAI	20
14	Ni(PPh_3_)_2_Cl_2_	Xylene	AgOTf	TBAI	0
15	Ni(PPh_3_)_2_Cl_2_	Xylene	—	TBAI	0
16	Ni(PPh_3_)_2_Cl_2_	Xylene	Cu(OAc)_2_	TBAI	0
17	Ni(PPh_3_)_2_Cl_2_	Xylene	Ag_2_CO_3_	NaI	0
18	Ni(PPh_3_)_2_Cl_2_	Xylene	Ag_2_CO_3_	TBAB	58
19	Ni(PPh_3_)_2_Cl_2_	Xylene	Ag_2_CO_3_	TBAF·4H_2_O	0
20	Ni(PPh_3_)_2_Cl_2_	Xylene	Ag_2_CO_3_	Me_4_NCl	Trace
21	Ni(PPh_3_)_2_Cl_2_	Xylene	Ag_2_CO_3_	—	0
22^c^	Ni(PPh_3_)_2_Cl_2_	Xylene	Ag_2_CO_3_	TBAI	64
23^d^	Ni(PPh_3_)_2_Cl_2_	Xylene	Ag_2_CO_3_	TBAI	61

a Reaction condition: compound 1a (0.1 mmol), 2a (0.2 mmol), catalyst (10 mol%), Na_2_CO_3_ (2 equiv.), oxidant (4 equiv.), additive (3 equiv.), solvent (2.0 mL), under air, 110 °C, 12 h.

b Estimated by ^1^H NMR spectroscopy using CH_2_Br_2_ as an internal reference, Q = quinolin-8-yl.

c Under Ar.

d Under O_2_.

**Scheme 3:**
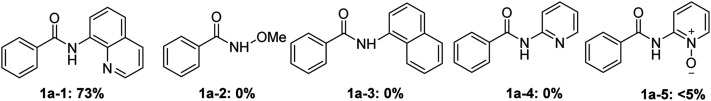
The effect of directing groups for the alkynylation/annulation reaction under standard reaction conditions.

Under the optimized conditions, we examined the applicability of the catalytic system for different directing groups, as shown in Scheme 3. Interestingly, *N*-(quinolin-8-yl) benzamide (1a-1) could promote the alkynylation/annulation reaction smoothly as well, delivering the corresponding isoindolinone in 73% yield, with high selectivity. Nevertheless, other common directing groups including the monodentate group, *N*-methoxybenzamide (1a-2), *N*-(naphthalen-1-yl) benzamide (1a-3), the bidentate coordinating groups, *N*-(pyridin-2-yl) benzamide (1a-4) and *N*-(pyridin-1-oxide-2-yl) benzamide (1a-5) were incapable of promoting the reaction. These control experiments showed the indispensable role of the 8-aminoquinoline moiety for this reaction.

**Scheme 4:**
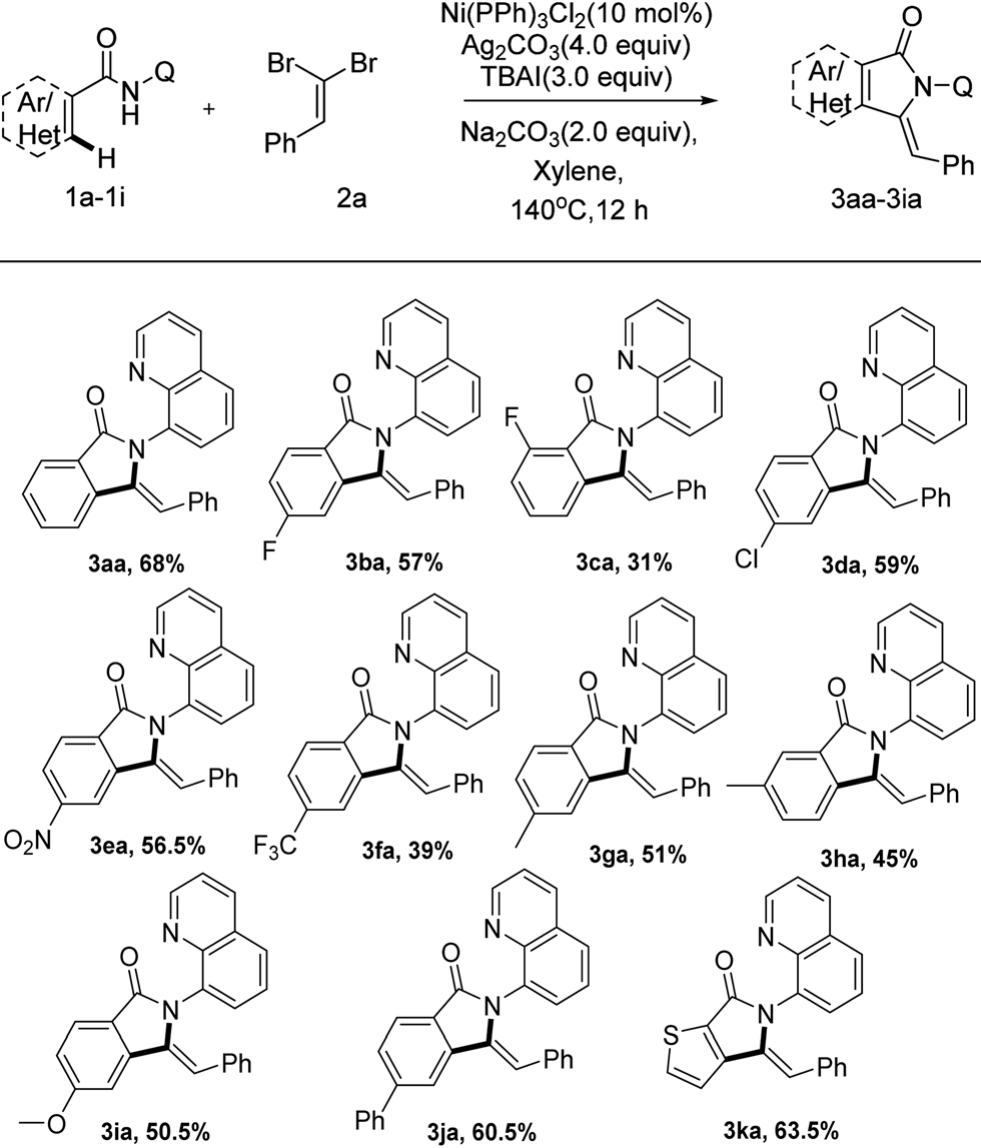
Scope of *N*-(quinolin-8-yl) benzamides^*a*,*b*^. ^*a*^Reaction condition: compound 1a (0.1 mmol), 2a (0.2 mmol), Ni(PPh_3_)_2_Cl_2_ (10 mol%), Na_2_CO_3_ (2 equiv.), Ag_2_CO_3_ (4 equiv.), TBAI (3 equiv.), solvent (2.0 mL), under air, 140 °C, 12 h. ^*b*^Isolated yield of 3 by flash column chromatography. Q = quinolin-8-yl, TBAI = tetrabutylammonium iodide.

Next, we set out to explore the scope of *N*-(quinolin-8-yl) benzamide partners as summarized in Scheme 4. To our delight, the reaction system could accelerate the tandem reaction of a wide array of *N*-(quinolin-8-yl) benzamides with (2,2-dibromovi-nyl)benzene, delivering a series of functionalized 3-methyleneisoindolin-1-ones in moderate to excellent yields. The positions of substituent on arenes had a remarkable effect on the transformation. While the substituent was installed on the *ortho*-position of the aromatic ring, the reaction did not proceed smoothly with only 31% yield (3ba–ca, 3ga–ha), which implicated that steric hindrance has a huge effect on the transformation. *N*-(Quinolin-8-yl) benzamides bearing the synthetically valuable groups, such as F, Cl, NO_2_ and CF_3_, were also tolerated as well in this transformation, delivering the corresponding products (3ba–fa) in moderate yields. Notably, the protocol was also compatible with heterocyclic substrates, liberating 3ka in acceptable yields (63.5%).

**Scheme 5:**
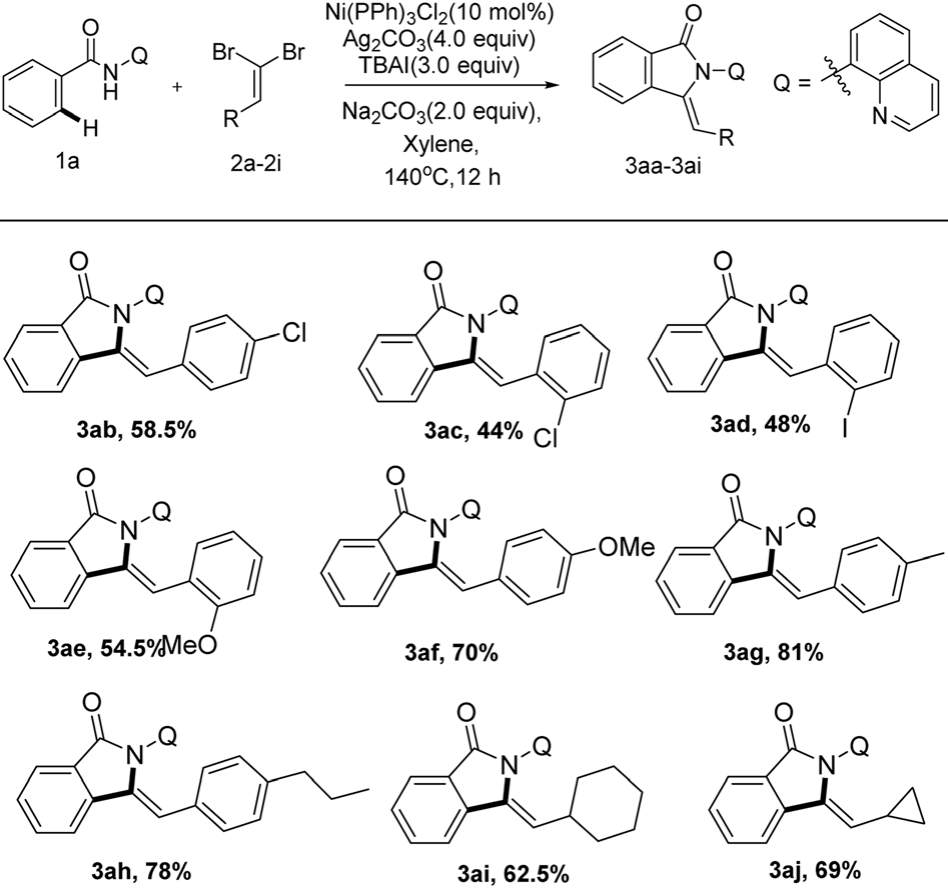
Scope of various dibromoalkenes^*a*,*b*^. ^*a*^Reaction condition: compound 1a (0.1 mmol), 2a (0.2 mmol), Ni(PPh_3_)_2_Cl_2_ (10 mol%), Na_2_CO_3_ (2 equiv.), Ag_2_CO_3_ (4 equiv.), TBAI (3 equiv.), solvent (2.0 mL), under air, 140 °C, 12 h. ^*b*^Isolated yield of 3 by flash column chromatography. Q = quinolin-8-yl, TBAI = tetrabutylammonium iodide.

Subsequently, we investigated a series of various dibromoalkenes under the optimized conditions as displayed in Scheme 5. Electron-rich and electron-deficient *gem*-dibromo olefins were all successfully engaged in this transformation (3ab–3ah). Dibromoalkenes bearing both electron-rich groups (Me, OMe and *N*-propyl substitutions) allowed better results furnishing alkynylation and cyclization products (3ae–3ah) between 54 to 81% isolated yields. The steric hindrance of the *ortho*-position on aromatic ring had an apparent effect on the reaction. Comparing with the *gem*-dibromoalkenes bearing substituents on the *para*-site (3ab, 58.5%; 3af, 70%), the *ortho*-substituted ones gave lower yields (3ac, 44%; 3ae, 54.5%). Additionally, we were pleased to notice that alkyl *gem*-dibromoalkenes were also tolerated under the catalytic system, delivering 3-methyleneisoindolin-1-ones in acceptable yields (3ai, 62.5%; 3aj, 69%), respectively.

**Scheme 6:**
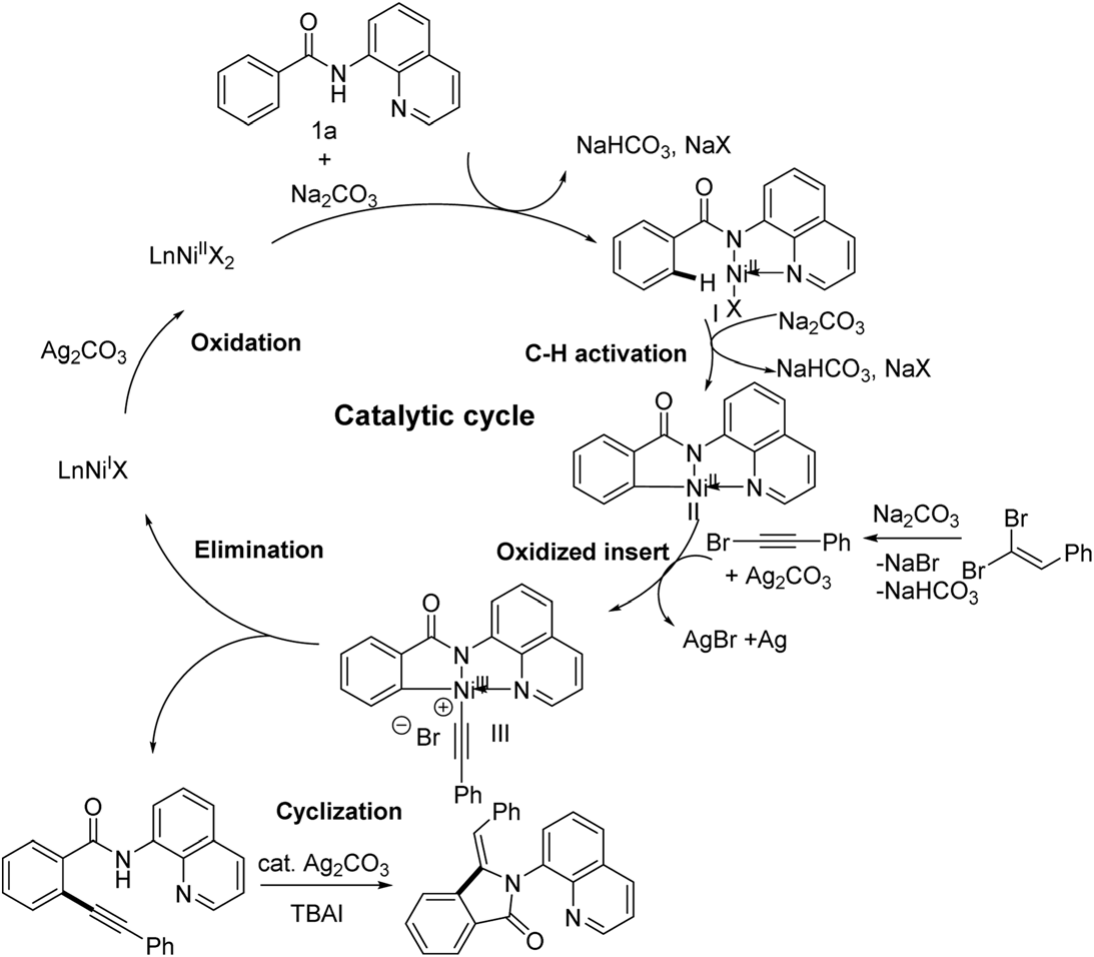
Plausible mechanism.

Very recently, the transition of metal-catalyzed tandem C–H activation and annulation of arene with terminal alkyne has been reported by many researchers, especially in which the Co(ii)/Co(iii) and Ni(i)/Ni(iii) catalytic cycle was proved.^9,22*a*^ On the basis of the above results of experiments and relevant literatures.^22^ We speculated that the reaction might also proceed through a Ni(i)/Ni(iii) catalytic process and the plausible reaction mechanism has been proposed in Scheme 6. The catalytic cycle initiates with the oxidation of Ni(ii) by Ag_2_CO_3_ to give Ni(iii) species, which activated the inert sp^2^ C–H bond of *N*-(quinolin-8-yl)benzamide (1a) to generate the key intermediate II. Subsequently, the attack of the corresponding bromoalkyne *via* dehydrobromination of the dibromoalkene into intermediate II to oxidize addition gives the essential intermediate III, which undergoes the reductive elimination to give the alkynylated product IV and liberate the Ni(ii) species. The oxidation of Ni(ii) to Ni(iii) by silver salts continues the cycle. And the *ortho*-alkynyl amide IV occurs rapidly intramolecular cyclization in the presence of Ag_2_CO_3_ and TBAI to give the 3-methyleneisoindolin-1-one products 3aa. Additionally, the possible pathway of a part of products is through the radical exchange of intermediate II with alkynyl radical and subsequent reductive elimination to give the alkynylated product IV through a Ni(iii)/Ni(i) mechanism.^22^

## Conclusions

In conclusion, we have developed an efficient, operationally simple, and scalable nickel/silver-catalyzed unactivated C sp^2^–H bond activation and cyclization for the synthesis of isoindolinones divergently with excellent selectivity from readily available *gem*-dibromoalkenes. This protocol enables operational convenience with good tolerance of various aromatic amides and dibromoalkenes. Furthermore, this practical methodology may provide insight into the development of transition metal catalyzed C–H functionalization and further complement existing synthetic methods.

## Experimental

### General information

Unless otherwise noted, all of the reagents were purchased from commercial suppliers and used without purification. All product mixtures were analyzed by thin layer chromatography glass-backed silica TLC plates with a fluorescent indicator from Branch of Qingdao Haiyang Chemical CO. LTD. UV-active compounds were detected with a UV lamp (*λ* = 254 nm). For flash column chromatography, silica gel (200–300 mesh) was used as stationary phase. ^1^H NMR spectra ere recorded on a Bruker Advance III 400 MHz spectrometer in deuterated chloroform. The chemical shifts (*δ*) are reported in parts per million relatives to tetramethylsilane. The multiplicities of signals are designated by the following abbreviations: s (singlet), d (doublet), t (triplet), q (quartet), m (multiplet). Coupling constants (*J*) are given in hertz. ^13^C NMR spectra were recorded using a 100 MHz spectrometer. The chemical shifts are reported relative to residual CHCl_3_ (*δ* C = 77.00 ppm). High resolution mass spectra (HRMS) were measured with a Waters Micromass GCT instrument, accurate masses were reported for the molecular ion ([M]^+^ or [M + H]^+^).

### General procedure for the preparation of carboxamides

To the solution of carboxylic acid (10 mmol) and 10 drops of DMF in 30 mL dry DCM at 0 °C, oxalyl chloride (20 mmol) was added dropwise under Ar. The mixture was then warm to r.t and stirred for another 5 h. The solvent was removed under vacuum to give crude acid cholid, which was used directly for next step without further purification.

To the mixture of 8-aminoquinoline (10 mmol) and Et_3_N (12 mmol) in dry DCM (30 mL) at 0 °C, the crude acid chloride obtained from previous step in 20 mL dry DCM was added dropwise. The mixture was then warm to r.t and stirred overnight. The reaction was quenched with H_2_O. The mixture was extracted, washed with saturated NaHCO_3_ solution. The combined organic layers were dried (MgSO_4_) and concentrated in vacuum and then purified by silica gel chromatography with a mixture of hexanes and ethyl acetate as the eluent to afford the corresponding amide products (reference: *Chem. Commun.*, 2015, **51**, 7863–7866).

### General procedure for the preparation of *gem*-dibromoalkenes

To an ice cooled stirred solution of aldehyde (5.0 mmol) and carbon tetrabromide (2.5 g, 7.5 mmol) in anhydrous CH_2_Cl_2_ (40 mL) was added slowly a solution of triphenylphosphine (4.0 g, 15.0 mmol) in dichloromethane (30 mL) by several portions. The reaction was monitored by TLC. After the reaction was complete, the mixture was diluted with hexane (100 mL) and purified directly by column chromatography on silica gel. If it is not specified, hexane was used as an eluent for the column chromatography (reference: *RSC Adv.*, 2014, **4**, 2322–2326).

### Typical procedure for copper(ii)/silver(i)-catalyzed sequential alkynylation and annulation of arenes with *gem*-dibromoalkenes

A mixture of *N*-(quinolin-8-yl) benzamides (1, 24.8 mg, 0.1 mmol), Ni(PPh_3_)_2_Cl_2_(10 mol%, 6.2 mg), Ag_2_CO_3_ (110.3 mg, 0.4 mmol), TBAI (110.8 mg, 0.3 mmol), *gem*-dibromoalkenes (2, 0.2 mmol) and xylene (2.0 mL) was added to a 25 mL open tube. The tube was stirred at 140 °C for 12 h under air. The reaction was monitored by TLC. After the reaction was complete, then the reaction mixture was cooled to room temperature, and the reaction solution was treated with dilute *p*-toluenesulfonic acid for half hour. A saturated solution of sodium bicarbonate (10.0 mL) was added to the reaction tube and the pH of the solution in the reaction tube is neutralized to about 6–7. The mixture was extracted with ethyl acetate (3 × 15 mL), and the organic phase was combined and dried over Na_2_SO_4_ and was concentrated *in vacuo*. Then the mixture was subjected to column chromatography on silica gel using petroleum ether/ethyl acetate = 2 : 1 as eluent to afford the desired products (3). And then calculated the yields.

### (*Z*)-3-Benzylidene-2-(quinolin-8-yl) isoindolin-1-one (3a)

^1^H NMR (400 MHz, CDCl_3_) *δ* 8.86 (dd, *J* = 4.2, 1.7 Hz, 1H), 7.98 (dd, *J* = 11.2, 4.6 Hz, 2H), 7.89 (d, *J* = 7.8 Hz, 1H), 7.69 (td, *J* = 7.6, 1.1 Hz, 1H), 7.60–7.54 (m, 2H), 7.48 (dd, *J* = 7.3, 1.4 Hz, 1H), 7.33–7.27 (m, 2H), 6.81 (s, 1H), 6.67 (dd, *J* = 11.1, 4.3 Hz, 1H), 6.58–6.51 (m, 4H). ^13^C NMR (101 MHz, CDCl_3_) *δ* 168.25, 150.47, 144.47, 138.77, 136.16, 135.92, 134.26, 133.62, 132.36, 130.14, 129.19, 128.94, 128.49, 128.38, 128.22, 126.39, 126.12, 125.75, 124.03, 121.33, 119.76, 107.49, 77.50, 77.18, 76.86. MS (ESI) *m*/*z*: 348.1 [M]^+^.

### (*Z*)-3-Benzylidene-5-fluoro-2-(quinolin-8-yl) isoindolin-1-one (3b)

^1^H NMR (400 MHz, CDCl_3_) *δ* 8.85 (dd, *J* = 4.2, 1.7 Hz, 1H), 8.85 (dd, *J* = 4.2, 1.7 Hz, 1H), 7.99–7.95 (m, 2H), 7.59 (dd, *J* = 8.2, 1.3 Hz, 1H), 7.54 (dd, *J* = 8.5, 2.1 Hz, 1H), 7.48 (dd, *J* = 7.4, 1.4 Hz, 1H), 7.33–7.27 (m, 3H), 6.75 (s, 1H), 6.69 (dt, *J* = 8.2, 4.1 Hz, 1H), 6.54 (d, *J* = 4.9 Hz, 4H). ^13^C NMR (101 MHz, CDCl_3_) *δ* 167.21, 150.49, 144.32, 135.95, 135.34, 133.99, 133.14, 130.09, 128.94, 128.58, 128.16, 126.43, 126.34, 126.21, 125.75, 124.50, 121.37, 117.16, 116.92, 108.49, 106.98, 106.73, 77.44, 77.12, 76.81. MS (ESI) *m*/*z*: 366.1 [M]^+^.

### (*Z*)-3-Benzylidene-5-chloro-2-(quinolin-8-yl) isoindolin-1-one (3c)

^1^H NMR (400 MHz, CDCl_3_) *δ* 8.84 (dd, *J* = 4.2, 1.7 Hz, 1H), 7.97 (dd, *J* = 8.3, 1.7 Hz, 1H), 7.92 (d, *J* = 8.1 Hz, 1H), 7.87 (d, *J* = 1.6 Hz, 1H), 7.59 (dd, *J* = 8.2, 1.4 Hz, 1H), 7.53 (dd, *J* = 8.1, 1.7 Hz, 1H), 7.48 (dd, *J* = 7.4, 1.4 Hz, 1H), 7.32–7.28 (m, 2H), 6.78 (s, 1H), 6.69 (dt, *J* = 8.8, 4.3 Hz, 1H), 6.54 (d, *J* = 4.5 Hz, 4H). ^13^C NMR (101 MHz, CDCl_3_) *δ* 167.27, 150.53, 144.35, 140.32, 138.84, 135.95, 135.19, 134.00, 133.19, 130.12, 129.60, 128.98, 128.66, 128.21, 126.79, 126.48, 126.41, 125.78, 125.37, 121.43, 120.18, 108.69, 77.48, 77.16, 76.84. MS (ESI) *m*/*z*: 382.0 [M]^+^.

### (*Z*)-3-Benzylidene-5-methyl-2-(quinolin-8-yl)isoindolin-1-one (3d)

^1^H NMR (400 MHz, CDCl_3_) *δ* 8.84 (dd, *J* = 4.2, 1.6 Hz, 1H), 7.96 (dd, *J* = 8.3, 1.6 Hz, 1H), 7.87 (d, *J* = 7.8 Hz, 1H), 7.68 (s, 1H), 7.57 (d, *J* = 8.2 Hz, 1H), 7.47 (dd, *J* = 7.3, 1.2 Hz, 1H), 7.37 (d, *J* = 7.2 Hz, 1H), 7.31–7.27 (m, 2H), 6.77 (s, 1H), 6.67 (t, *J* = 6.4 Hz, 1H), 6.56–6.49 (m, 4H), 2.55 (s, 3H). ^13^C NMR (101 MHz, CDCl_3_) *δ* 168.33, 150.41, 144.47, 143.04, 139.14, 136.24, 135.90, 134.36, 133.73, 130.32, 130.13, 129.60, 128.92, 128.37, 128.21, 126.35, 126.01, 125.74, 123.87, 121.27, 120.07, 107.04, 77.46, 77.14, 76.83, 22.30. MS (ESI) *m*/*z*: 362.1 [M]^+^.

### (*Z*)-3-Benzylidene-5-methoxy-2-(quinolin-8-yl) isoindolin-1-one (3e)

^1^H NMR (400 MHz, CDCl_3_) *δ* 8.84 (dd, *J* = 4.2, 1.7 Hz, 1H), 7.96 (dd, *J* = 8.3, 1.7 Hz, 1H), 7.89 (d, *J* = 8.4 Hz, 1H), 7.56 (dd, *J* = 8.2, 1.3 Hz, 1H), 7.47 (dd, *J* = 7.4, 1.4 Hz, 1H), 7.33 (d, *J* = 2.1 Hz, 1H), 7.30–7.26 (m, 2H), 7.09 (dd, *J* = 8.4, 2.2 Hz, 1H), 6.74 (s, 1H), 6.67 (ddd, *J* = 8.7, 4.7, 1.9 Hz, 1H), 6.57–6.50 (m, 4H), 3.96 (s, 3H). ^13^C NMR (101 MHz, CDCl_3_) *δ* 168.05, 163.62, 150.40, 144.52, 141.05, 136.20, 135.91, 134.41, 133.63, 130.16, 128.95, 128.34, 128.23, 126.38, 126.09, 125.76, 125.56, 121.38, 121.27, 116.58, 107.12, 103.81, 77.48, 77.16, 76.84, 55.94. MS (ESI) *m*/*z*: 378.1 [M]^+^.

### (*Z*)-3-Benzylidene-6-methyl-2-(quinolin-8-yl) isoindolin-1-one (3f)

^1^H NMR (400 MHz, CDCl_3_) *δ* 8.84 (dd, *J* = 4.2, 1.7 Hz, 1H), 7.96 (dd, *J* = 8.3, 1.7 Hz, 1H), 7.79–7.78 (m, 1H), 7.76 (d, *J* = 7.9 Hz, 1H), 7.57 (dd, *J* = 8.2, 1.4 Hz, 1H), 7.50–7.47 (m, 1H), 7.46 (dd, *J* = 7.4, 1.4 Hz, 1H), 7.29 (dd, *J* = 4.4, 3.9 Hz, 1H), 7.26 (d, *J* = 8.2 Hz, 1H), 6.74 (s, 1H), 6.69–6.64 (m, 1H), 6.56–6.49 (m, 4H), 2.51 (s, 3H). ^13^C NMR (101 MHz, CDCl_3_) *δ* 168.42, 150.45, 144.56, 139.52, 136.40, 136.30, 135.90, 134.44, 133.83, 133.44, 130.17, 128.96, 128.63, 128.41, 128.28, 126.39, 126.00, 125.76, 124.12, 121.31, 119.59, 106.74, 77.48, 77.16, 76.84, 21.72. MS (ESI) *m*/*z*: 362.1 [M]^+^.

### (*Z*)-3-Benzylidene-5-phenyl-2-(quinolin-8-yl) isoindolin-1-one (3g)

^1^H NMR (400 MHz, CDCl_3_) *δ* 8.86 (dd, *J* = 4.2, 1.7 Hz, 1H), 8.07–8.04 (m, 2H), 7.97 (dd, *J* = 8.3, 1.7 Hz, 1H), 7.78 (dd, *J* = 7.9, 1.4 Hz, 1H), 7.72 (dd, *J* = 8.1, 1.0 Hz, 2H), 7.59 (dd, *J* = 8.2, 1.3 Hz, 1H), 7.54–7.50 (m, 3H), 7.45 (d, *J* = 7.2 Hz, 1H), 7.33–7.29 (m, 2H), 6.88 (s, 1H), 6.71–6.65 (m, 1H), 6.58–6.51 (m, 4H). ^13^C NMR (101 MHz, CDCl_3_) *δ* 168.10, 150.49, 145.82, 144.48, 140.81, 139.42, 136.25, 135.95, 135.12, 134.33, 133.64, 130.23, 129.14, 129.00, 128.58, 128.51, 128.27, 127.69, 127.28, 126.43, 126.16, 125.81, 124.47, 121.36, 118.54, 107.58, 77.48, 77.16, 76.84. MS (ESI) *m*/*z*: 424.1 [M]^+^.

### (*Z*)-3-Benzylidene-5-nitro-2-(quinolin-8-yl) isoindolin-1-one (3h)

^1^H NMR (400 MHz, CDCl_3_) *δ* 8.83 (dd, *J* = 4.2, 1.7 Hz, 1H), 8.76 (d, *J* = 1.8 Hz, 1H), 8.42 (dd, *J* = 8.3, 1.9 Hz, 1H), 8.15 (d, *J* = 8.3 Hz, 1H), 8.00 (dd, *J* = 8.3, 1.7 Hz, 1H), 7.63 (dd, *J* = 8.2, 1.3 Hz, 1H), 7.51 (dd, *J* = 7.4, 1.4 Hz, 1H), 7.36–7.31 (m, 2H), 6.97 (s, 1H), 6.76–6.70 (m, 1H), 6.56 (d, *J* = 4.7 Hz, 4H). ^13^C NMR (101 MHz, CDCl_3_) *δ* 166.11, 150.86, 150.64, 144.02, 139.58, 136.09, 134.65, 133.49, 132.79, 132.59, 130.03, 129.03, 128.17, 126.81, 126.59, 125.82, 125.28, 124.09, 121.61, 115.70, 110.61, 77.48, 77.16, 76.84. MS (ESI) *m*/*z*: 393.1 [M]^+^.

### (*Z*)-3-(4-Chlorobenzylidene)-2-(quinolin-8-yl) isoindolin-1-one (3i)

^1^H NMR (400 MHz, CDCl_3_) *δ* 8.83 (dd, *J* = 4.2, 1.7 Hz, 1H), 8.04–7.97 (m, 2H), 7.87 (d, *J* = 7.8 Hz, 1H), 7.69 (dd, *J* = 13.6, 7.3 Hz, 2H), 7.58 (t, *J* = 7.5 Hz, 1H), 7.51 (d, *J* = 7.3 Hz, 1H), 7.38–7.30 (m, 2H), 6.71 (s, 1H), 6.50–6.43 (m, 4H). ^13^C NMR (101 MHz, CDCl_3_) *δ* 168.13, 150.54, 144.38, 138.52, 136.86, 136.01, 134.17, 132.49, 132.09, 131.91, 130.25, 129.43, 129.38, 129.00, 128.62, 128.39, 126.36, 125.89, 124.14, 121.54, 119.81, 105.91, 77.48, 77.16, 76.84. MS (ESI) *m*/*z*: 382.0 [M]^+^.

### (*Z*)-3-(2-Chlorobenzylidene)-2-(quinolin-8-yl) isoindolin-1-one (3j)

^1^H NMR (400 MHz, CDCl_3_) *δ* 8.89 (dd, *J* = 4.2, 1.6 Hz, 1H), 8.02–7.92 (m, 3H), 7.71 (t, *J* = 7.6 Hz, 1H), 7.62–7.54 (m, 3H), 7.34–7.28 (m, 2H), 6.86 (d, *J* = 8.0 Hz, 1H), 6.71 (s, 1H), 6.62 (t, *J* = 7.7 Hz, 1H), 6.35 (d, *J* = 7.7 Hz, 1H), 6.14 (t, *J* = 7.5 Hz, 1H). ^13^C NMR (101 MHz, CDCl_3_) *δ* 168.24, 150.79, 144.49, 138.40, 137.24, 135.85, 133.82, 133.01, 132.50, 132.44, 130.49, 130.00, 129.52, 128.76, 128.62, 128.54, 127.89, 127.72, 125.77, 124.34, 124.09, 121.39, 120.13, 104.37, 77.48, 77.16, 76.84. MS (ESI) *m*/*z*: 382.0 [M]^+^.

### (*Z*)-3-(2-Iodobenzylidene)-2-(quinolin-8-yl) isoindolin-1-one (3k)

^1^H NMR (400 MHz, CDCl_3_) *δ* 8.94 (dd, *J* = 3.3, 0.8 Hz, 1H), 8.02–7.97 (m, 2H), 7.95 (d, *J* = 7.8 Hz, 1H), 7.71 (t, *J* = 7.6 Hz, 1H), 7.60 (d, *J* = 8.1 Hz, 1H), 7.58–7.55 (m, 1H), 7.54 (s, 1H), 7.35 (d, *J* = 6.8 Hz, 1H), 7.33–7.26 (m, 2H), 6.58 (s, 1H), 6.39–6.33 (m, 2H), 6.21 (t, *J* = 7.5 Hz, 1H). ^13^C NMR (101 MHz, CDCl_3_) *δ* 168.23, 150.99, 144.46, 138.38, 137.86, 137.25, 136.58, 135.93, 133.80, 132.51, 130.14, 129.87, 129.50, 128.76, 128.64, 128.48, 127.81, 125.79, 125.75, 124.12, 121.42, 120.03, 110.49, 99.83, 77.48, 77.16, 76.84. MS (ESI) *m*/*z*: 474.0 [M]^+^.

### (*Z*)-3-(2-Methoxybenzylidene)-2-(quinolin-8-yl) isoindolin-1-one (3l)

^1^H NMR (400 MHz, CDCl_3_) *δ* 8.86 (dd, *J* = 4.2, 1.7 Hz, 1H), 7.99–7.98 (m, 1H), 7.97–7.95 (m, 1H), 7.92 (d, *J* = 7.8 Hz, 1H), 7.67 (td, *J* = 7.6, 1.1 Hz, 1H), 7.55 (td, *J* = 7.9, 4.1 Hz, 2H), 7.48 (dd, *J* = 7.3, 1.4 Hz, 1H), 7.31–7.27 (m, 2H), 6.76 (s, 1H), 6.67 (ddd, *J* = 8.5, 7.6, 1.2 Hz, 1H), 6.30 (t, *J* = 8.2 Hz, 2H), 5.92 (td, *J* = 7.4, 0.7 Hz, 1H), 3.58 (s, 3H). ^13^C NMR (101 MHz, CDCl_3_) *δ* 168.33, 156.18, 150.34, 144.60, 138.77, 136.38, 135.95, 134.10, 132.22, 130.05, 130.00, 129.00, 128.79, 128.46, 128.39, 127.93, 125.52, 123.91, 122.52, 121.11, 120.10, 118.57, 108.84, 103.92, 77.48, 77.16, 76.84, 55.03. MS (ESI) *m*/*z*: 378.1 [M]^+^.

### (*Z*)-3-(4-Methylbenzylidene)-2-(quinolin-8-yl) isoindolin-1-one (3n)

^1^H NMR (400 MHz, CDCl_3_) *δ* 8.83 (dd, *J* = 4.1, 1.5 Hz, 1H), 7.97 (t, *J* = 7.8 Hz, 2H), 7.85 (d, *J* = 7.8 Hz, 1H), 7.65 (t, *J* = 7.6 Hz, 1H), 7.59–7.51 (m, 2H), 7.45 (d, *J* = 7.4 Hz, 1H), 7.30–7.25 (m, 2H), 6.78 (s, 1H), 6.42 (d, *J* = 7.8 Hz, 2H), 6.31 (d, *J* = 7.8 Hz, 2H), 1.99 (s, 3H). ^13^C NMR (101 MHz, CDCl_3_) *δ* 168.17, 150.38, 144.52, 138.81, 135.82, 135.72, 134.38, 132.25, 130.53, 130.07, 128.99, 128.91, 128.28, 128.14, 128.06, 127.00, 125.72, 123.93, 121.29, 119.67, 107.67, 77.48, 77.16, 76.84, 20.95. MS (ESI) *m*/*z*: 362.1 [M]^+^.

### (*Z*)-3-(4-Propylbenzylidene)-2-(quinolin-8-yl)isoindolin-1-one (3o)

^1^H NMR (400 MHz, CDCl_3_) *δ* 8.83 (dd, *J* = 4.2, 1.7 Hz, 1H), 8.00–7.92 (m, 2H), 7.86 (d, *J* = 7.8 Hz, 1H), 7.66 (td, *J* = 7.6, 1.1 Hz, 1H), 7.54 (dd, *J* = 7.8, 6.9 Hz, 2H), 7.47 (dd, *J* = 7.4, 1.3 Hz, 1H), 7.30–7.26 (m, 2H), 6.79 (s, 1H), 6.45 (d, *J* = 7.9 Hz, 2H), 6.32 (d, *J* = 8.0 Hz, 2H), 2.22 (t, *J* = 7.5 Hz, 2H), 1.42–1.32 (m, 2H), 0.80 (t, *J* = 7.3 Hz, 3H). ^13^C NMR (101 MHz, CDCl_3_) *δ* 168.18, 150.40, 144.54, 140.56, 138.80, 135.89, 135.81, 134.38, 132.26, 130.89, 130.11, 129.02, 128.88, 128.32, 128.08, 126.53, 125.72, 123.98, 121.25, 119.70, 107.73, 77.48, 77.16, 76.84, 37.53, 24.47, 13.80. MS (ESI) *m*/*z*: 390.1 [M]^+^.

### (*Z*)-4-Benzylidene-5-(quinolin-8-yl)-4,5-dihydro-6*H*-thieno[2,3-c] pyrrol-6-one (3p)

^1^H NMR (400 MHz, CDCl_3_) *δ* 8.92 (dd, *J* = 4.2, 1.7 Hz, 1H), 8.08 (dd, *J* = 8.3, 1.7 Hz, 1H), 7.76 (d, *J* = 5.2 Hz, 1H), 7.72 (dd, *J* = 8.2, 1.3 Hz, 1H), 7.51 (dd, *J* = 7.3, 1.4 Hz, 1H), 7.44–7.39 (m, 1H), 7.37 (dd, *J* = 8.3, 4.2 Hz, 1H), 7.29 (d, *J* = 5.2 Hz, 1H), 7.09 (d, *J* = 6.9 Hz, 2H), 7.04–6.99 (m, 1H), 6.94 (t, *J* = 7.4 Hz, 2H), 6.79 (s, 1H). ^13^C NMR (101 MHz, CDCl_3_) *δ* 159.32, 151.10, 146.16, 145.52, 144.79, 136.93, 136.37, 136.27, 134.06, 130.99, 129.48, 129.00, 128.96, 128.87, 128.12, 127.41, 125.89, 124.66, 121.68, 104.66, 77.48, 77.16, 76.84. MS (ESI) *m*/*z*: 354.0 [M]^+^.

### (*Z*)-3-(Cyclohexylmethylene)-2-(quinolin-8-yl) isoindolin-1-one (3q)

^1^H NMR (400 MHz, CDCl_3_) *δ* 8.89 (dd, *J* = 4.2, 1.7 Hz, 1H), 8.24 (dd, *J* = 8.3, 1.7 Hz, 1H), 8.00–7.92 (m, 2H), 7.80 (dd, *J* = 7.3, 1.4 Hz, 1H), 7.72 (d, *J* = 7.8 Hz, 1H), 7.69–7.64 (m, 1H), 7.60 (td, *J* = 7.6, 1.1 Hz, 1H), 7.48 (td, *J* = 7.5, 0.8 Hz, 1H), 7.42 (dd, *J* = 8.3, 4.2 Hz, 1H), 5.45 (d, *J* = 10.7 Hz, 1H), 1.72 (dt, *J* = 14.6, 6.7 Hz, 1H), 1.48–1.42 (m, 1H), 1.41–1.35 (m, 1H), 1.26 (d, *J* = 7.0 Hz, 2H), 1.23–1.17 (m, 1H), 1.15–1.07 (m, 1H), 0.93–0.85 (m, 2H), 0.74 (ddd, *J* = 12.0, 10.8, 3.5 Hz, 2H). ^13^C NMR (101 MHz, CDCl_3_) *δ* 168.48, 151.33, 145.68, 138.84, 136.21, 135.72, 134.04, 131.93, 130.67, 129.30, 129.27, 128.42, 128.39, 126.35, 123.76, 121.92, 119.40, 115.12, 77.48, 77.16, 76.84, 35.28, 33.32, 33.15, 25.69, 25.57, 25.45. MS (ESI) *m*/*z*: 353.1 [M]^+^.

### (*Z*)-3-(Cyclopropylmethylene)-2-(quinolin-8-yl) isoindolin-1-one (3r)

^1^H NMR (400 MHz, CDCl_3_) *δ* 8.92 (dd, *J* = 4.2, 1.7 Hz, 1H), 8.19 (dd, *J* = 8.3, 1.7 Hz, 1H), 7.93 (d, *J* = 7.6 Hz, 1H), 7.90 (dd, *J* = 8.2, 1.3 Hz, 1H), 7.85 (dd, *J* = 7.3, 1.3 Hz, 1H), 7.66–7.62 (m, 2H), 7.57 (td, *J* = 7.7, 0.9 Hz, 1H), 7.46 (t, *J* = 7.7 Hz, 1H), 7.41 (dd, *J* = 8.3, 4.2 Hz, 1H), 5.13–5.06 (m, 1H), 0.40–0.32 (m, 2H), 0.28 (dd, *J* = 9.2, 6.5 Hz, 1H), 0.11 (ddd, *J* = 11.9, 8.6, 3.6 Hz, 1H), −0.04 (tdd, *J* = 8.5, 6.4, 4.5 Hz, 1H). ^13^C NMR (101 MHz, CDCl_3_) *δ* 168.07, 151.23, 145.52, 138.15, 136.16, 135.46, 135.28, 131.89, 130.66, 129.20, 129.14, 128.16, 127.82, 126.26, 123.74, 121.82, 118.97, 113.93, 77.48, 77.16, 76.84, 8.76, 8.66. MS (ESI) *m*/*z*: 312.1 [M]^+^.

## Conflicts of interest

There are no conflicts to declare.

## Supplementary Material

RA-008-C8RA03278E-s001
