# Supplementary material for: Nickel(ii)-catalyzed tandem C(sp2)–H bond activation and annulation of arenes with gem-dibromoalkenes
Source: RSC Adv. 2018 Aug 13;8(50):28668–75. doi: 10.1039/c8ra03278e (PMC9092601; doi:10.1039/c8ra03278e)

Electronic Supplementary Material (ESI) for RSC Advances.
This journal is © The Royal Society of Chemistry 2018

Supporting Information

Nickel(Ⅱ)-Catalyzed Tandem C(sp²) –H bonds activation and Annulation of Arenes with gem-dibromoalkenes

Yun Shi,[a] Meng-Sheng Li,[a] Fangdong Zhang,[a] and Baohua Chen*[a]

College of Chemistry and Chemical Engineering, and State Key Laboratory of Applied Organic Chemistry, Lanzhou University, Lanzhou, 730000, China

E-mail: chbh@lzu.edu.cn

1. **General information** S2
2. **General procedure for the preparation of Carboxamides** S2
3. **General procedure for the preparation of gem-dibromoalkenes** S2
4. **Typical procedure for copper(II)/silver(I)-catalyzed sequential alkynylation and annulation of Arenes with *gem*-dibromoalkenes** S3
5. **^{1}H NMR and ^{13}C NMR spectra of products 3** S9

1. General information

Unless otherwise noted, all of the reagents were purchased from commercial suppliers and used without purification. All product mixtures were analyzed by thin layer chromatography glass-backed silica TLC plates with a fluorescent indicator from Branch of Qingdao Haiyang Chemical CO. LTD. UV-active compounds were detected with a UV lamp (λ = 254 nm). For flash column chromatography, silica gel (200-300 mesh) was used as stationary phase. ^{1}H NMR spectra ere recorded on a Bruker Advance III 400 MHz spectrometer in deuterated chloroform. The chemical shifts (δ) are reported in parts per million relative to tetramethylsilane. The multiplicities of signals are designated by the following abbreviations: s (singlet), d (doublet), t (triplet), q (quartet), m (multiplet). Coupling constants (J) are given in hertz. ^{13}C NMR spectra were recorded using a 100 MHz spectrometer. The chemical shifts are reported relative to residual $CHCl_3$ (δ C= 77.00 ppm). High resolution mass spectra (HRMS) were measured with a Waters Micromass GCT instrument, accurate masses were reported for the molecular ion ($[M]^+$ or $[M+H]^+$).

2. General procedure for the preparation of Carboxamides

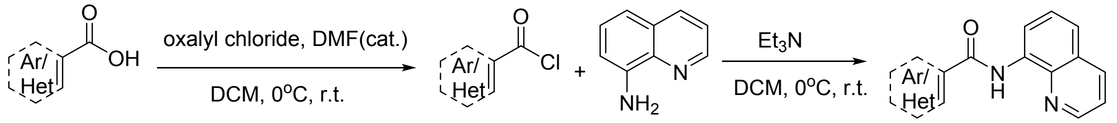

To the solution of carboxylic acid (10 mmol) and 10 drops of DMF in 30mL dry DCM at 0℃, oxalyl chloride (20 mmol) was added dropwise under Ar. The mixture was then warm to r.t and stired for another 5h. The solvent was removed under vacuum to give crude acid cholid, which was used directly for next step without further purification.

To the mixuture of 8-aminoquinoline (10 mmol) and Et_3N (12 mmol) in dry DCM (30 mL) at 0℃, the crude acid chloride obtained from previous step in 20 mL dry DCM was added dropwise. The mixture was then warm to r.t and stirred overnight. The reaction was quenched with H_2O. The mixture was extracted, washed with saturated $NaHCO_3$ solution. The combined organic layers were dried ($MgSO_4$), and concentrated in vacuum and then purified by silica gel chromatography with a mixture of hexanes and ethyl acetate as the eluent to afford the corresponding amide products.

(Reference: ***Chem. Commun.,*** 2015, 51, 7863-7866)

3. General procedure for the preparation of gem-dibromoalkenes

To an ice cooled stirred solution of aldehyde (5.0 mmol) and carbon tetrabromide (2.5g, 7.5 mmol) in anhydrous CH_2Cl_2 (40 mL) was added slowly a solution of triphenylphosphine (4.0g, 15.0 mmol) in dichloromethane (30 mL) by several portions. The reaction was monitored by TLC. After the reaction was complete, the mixture was diluted with hexane (100 mL) and purified directly by column

chromatography on silica gel. If it is not specified, hexane was used as an eluent for the column chromatography. (Reference: ***RSC Adv.***, 2014, **4**, 2322-2326)

4. **Typical procedure for copper(II)/silver(I)-catalyzed sequential alkynylation and annulation of Arenes with *gem*-dibromoalkenes**

A mixture of *N*-(quinolin-8-yl)benzamides (**1**, 24.8 mg, 0.1 mmol), $Ni(PPh_3)_2Cl_2$(10 mol%, 6.2 mg), Ag2CO3 (110.3 mg, 0.4 mmol), TBAI (110.8 mg, 0.3 mmol), gem-dibromoalkenes (**2**, 0.2 mmol) and Xylene (2.0 mL) was added to a 25 mL open tube. The tube was stirred at 140 °C for 12 h under air. The reaction was monitored by TLC. After the reaction was complete, then the reaction mixture was cooled to room temperature, and the reaction solution was treated with dilute *p*-toluenesulfonic acid for half hour. A saturated solution of sodium bicarbonate (10.0 mL) was added to the reaction tube and the pH of the solution in the reaction tube is neutralized to about 6-7. The mixture was extracted with ethyl acetate (3 x 15 mL), and the organic phase was combined and dried over Na_2SO_4 and was concentrated in vacuo. Then the mixture was subjected to column chromatography on silica gel using petroleum ether /ethyl acetate = 2:1 as eluent to afford the desired products (**3**). And then calculated the yields.

(*Z*)-3-benzylidene-2-(quinolin-8-yl)isoindolin-1-one(3a)

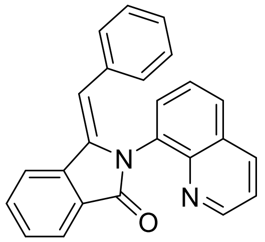

^{1}H NMR (400 MHz, $CDCl_3$) δ 8.86 (dd, *J* = 4.2, 1.7 Hz, 1H), 7.98 (dd, *J* = 11.2, 4.6 Hz, 2H), 7.89 (d, *J* = 7.8 Hz, 1H), 7.69 (td, *J* = 7.6, 1.1 Hz, 1H), 7.60 – 7.54 (m, 2H), 7.48 (dd, *J* = 7.3, 1.4 Hz, 1H), 7.33 – 7.27 (m, 2H), 6.81 (s, 1H), 6.67 (dd, *J* = 11.1, 4.3 Hz, 1H), 6.58 – 6.51 (m, 4H). ^{13}C NMR (101 MHz, $CDCl_3$) δ 168.25, 150.47, 144.47, 138.77, 136.16, 135.92, 134.26, 133.62, 132.36, 130.14, 129.19, 128.94, 128.49, 128.38, 128.22, 126.39, 126.12, 125.75, 124.03, 121.33, 119.76, 107.49, 77.50, 77.18, 76.86. MS(ESI) *m/z*: 348.1 $[M]^+$.

(*Z*)-3-benzylidene-5-fluoro-2-(quinolin-8-yl)isoindolin-1-one(3b)

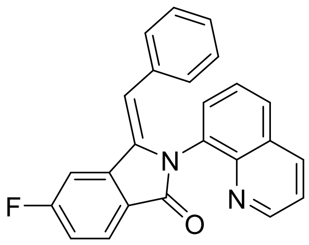

^{1}H NMR (400 MHz, $CDCl_3$) δ 8.85 (dd, *J* = 4.2, 1.7 Hz, 1H), 8.85 (dd, *J* = 4.2, 1.7 Hz, 1H), 7.99 – 7.95 (m, 2H), 7.59 (dd, *J* = 8.2, 1.3 Hz, 1H), 7.54 (dd, *J* = 8.5, 2.1 Hz, 1H), 7.48 (dd, *J* = 7.4, 1.4 Hz, 1H), 7.33 – 7.27 (m, 3H), 6.75 (s, 1H), 6.69 (dt, *J* = 8.2, 4.1 Hz, 1H), 6.54 (d, *J* = 4.9 Hz, 4H). ^{13}C NMR (101 MHz, $CDCl_3$) δ 167.21, 150.49, 144.32, 135.95, 135.34, 133.99, 133.14, 130.09, 128.94, 128.58, 128.16, 126.43, 126.34, 126.21, 125.75, 124.50, 121.37, 117.16, 116.92, 108.49, 106.98, 106.73, 77.44, 77.12, 76.81. MS(ESI) *m/z*: 366.1 $[M]^+$.

(*Z*)-3-benzylidene-5-chloro-2-(quinolin-8-yl)isoindolin-1-one(3c)

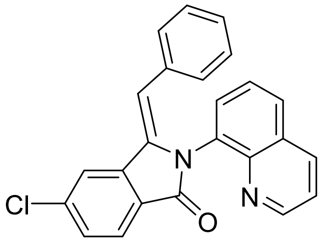

^{1}H NMR (400 MHz, $CDCl_3$) δ 8.84 (dd, *J* = 4.2, 1.7 Hz, 1H), 7.97 (dd, *J* = 8.3, 1.7 Hz, 1H), 7.92 (d, *J* = 8.1 Hz, 1H), 7.87 (d, *J* = 1.6 Hz, 1H), 7.59 (dd, *J* = 8.2, 1.4 Hz, 1H), 7.53 (dd, *J* = 8.1, 1.7 Hz, 1H), 7.48 (dd, *J* = 7.4, 1.4 Hz, 1H), 7.32 – 7.28 (m, 2H), 6.78 (s, 1H), 6.69 (dt, *J* = 8.8, 4.3 Hz, 1H), 6.54 (d, *J* = 4.5 Hz, 4H). ^{13}C NMR (101 MHz, $CDCl_3$) δ 167.27, 150.53, 144.35, 140.32, 138.84, 135.95, 135.19, 134.00, 133.19, 130.12, 129.60, 128.98, 128.66, 128.21, 126.79, 126.48, 126.41, 125.78, 125.37, 121.43, 120.18, 108.69, 77.48, 77.16, 76.84. MS(ESI) *m/z*: 382.0 $[M]^+$.

(*Z*)-3-benzylidene-5-methyl-2-(quinolin-8-yl)isoindolin-1-one(3d)

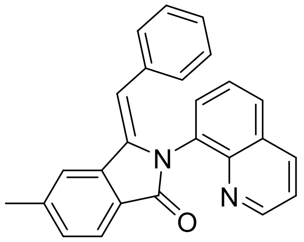

^{1}H NMR (400 MHz, $CDCl_3$) δ 8.84 (dd, *J* = 4.2, 1.6 Hz, 1H), 7.96 (dd, *J* = 8.3, 1.6 Hz, 1H), 7.87 (d, *J* = 7.8 Hz, 1H), 7.68 (s, 1H), 7.57 (d, *J* = 8.2 Hz, 1H), 7.47 (dd, *J* = 7.3, 1.2 Hz, 1H), 7.37 (d, *J* = 7.2 Hz, 1H), 7.31 – 7.27 (m, 2H), 6.77 (s, 1H), 6.67 (t, *J* = 6.4 Hz, 1H), 6.56 – 6.49 (m, 4H), 2.55 (s, 3H). ^{13}C NMR (101 MHz, $CDCl_3$) δ 168.33, 150.41, 144.47, 143.04, 139.14, 136.24, 135.90, 134.36, 133.73, 130.32, 130.13, 129.60, 128.92,

128.37, 128.21, 126.35, 126.01, 125.74, 123.87, 121.27, 120.07, 107.04, 77.46, 77.14, 76.83, 22.30. MS(ESI) *m/z*: 362.1 [M]$^+$.

(*Z*)-3-benzylidene-5-methoxy-2-(quinolin-8-yl)isoindolin-1-one(3e)

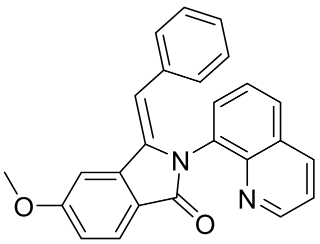

^{1}H NMR (400 MHz, $CDCl_3$) δ 8.84 (dd, *J* = 4.2, 1.7 Hz, 1H), 7.96 (dd, *J* = 8.3, 1.7 Hz, 1H), 7.89 (d, *J* = 8.4 Hz, 1H), 7.56 (dd, *J* = 8.2, 1.3 Hz, 1H), 7.47 (dd, *J* = 7.4, 1.4 Hz, 1H), 7.33 (d, *J* = 2.1 Hz, 1H), 7.30 – 7.26 (m, 2H), 7.09 (dd, *J* = 8.4, 2.2 Hz, 1H), 6.74 (s, 1H), 6.67 (ddd, *J* = 8.7, 4.7, 1.9 Hz, 1H), 6.57 – 6.50 (m, 4H), 3.96 (s, 3H). ^{13}C NMR (101 MHz, $CDCl_3$) δ 168.05, 163.62, 150.40, 144.52, 141.05, 136.20, 135.91, 134.41, 133.63, 130.16, 128.95, 128.34, 128.23, 126.38, 126.09, 125.76, 125.56, 121.38, 121.27, 116.58, 107.12, 103.81, 77.48, 77.16, 76.84, 55.94. MS(ESI) *m/z*: 378.1 [M]$^+$.

(*Z*)-3-benzylidene-6-methyl-2-(quinolin-8-yl)isoindolin-1-one(3f)

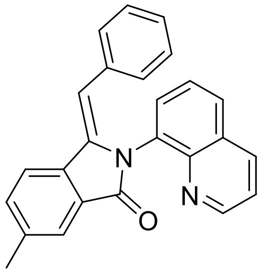

^{1}H NMR (400 MHz, $CDCl_3$) δ 8.84 (dd, *J* = 4.2, 1.7 Hz, 1H), 7.96 (dd, *J* = 8.3, 1.7 Hz, 1H), 7.79 – 7.78 (m, 1H), 7.76 (d, *J* = 7.9 Hz, 1H), 7.57 (dd, *J* = 8.2, 1.4 Hz, 1H), 7.50 – 7.47 (m, 1H), 7.46 (dd, *J* = 7.4, 1.4 Hz, 1H), 7.29 (dd, *J* = 4.4, 3.9 Hz, 1H), 7.26 (d, *J* = 8.2 Hz, 1H), 6.74 (s, 1H), 6.69 – 6.64 (m, 1H), 6.56 – 6.49 (m, 4H), 2.51 (s, 3H). ^{13}C NMR (101 MHz, $CDCl_3$) δ 168.42, 150.45, 144.56, 139.52, 136.40, 136.30, 135.90, 134.44, 133.83, 133.44, 130.17, 128.96, 128.63, 128.41, 128.28, 126.39, 126.00, 125.76, 124.12, 121.31, 119.59, 106.74, 77.48, 77.16, 76.84, 21.72. MS(ESI) *m/z*: 362.1 [M]$^+$.

(*Z*)-3-benzylidene-5-phenyl-2-(quinolin-8-yl)isoindolin-1-one(3g)

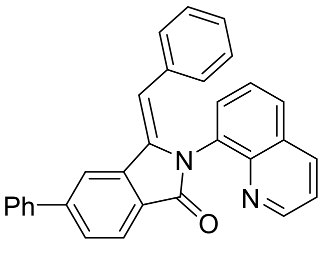

^{1}H NMR (400 MHz, $CDCl_3$) δ 8.86 (dd, J = 4.2, 1.7 Hz, 1H), 8.07 – 8.04 (m, 2H), 7.97 (dd, J = 8.3, 1.7 Hz, 1H), 7.78 (dd, J = 7.9, 1.4 Hz, 1H), 7.72 (dd, J = 8.1, 1.0 Hz, 2H), 7.59 (dd, J = 8.2, 1.3 Hz, 1H), 7.54 – 7.50 (m, 3H), 7.45 (d, J = 7.2 Hz, 1H), 7.33 – 7.29 (m, 2H), 6.88 (s, 1H), 6.71 – 6.65 (m, 1H), 6.58 – 6.51 (m, 4H). ^{13}C NMR (101 MHz, $CDCl_3$) δ 168.10, 150.49, 145.82, 144.48, 140.81, 139.42, 136.25, 135.95, 135.12, 134.33, 133.64, 130.23, 129.14, 129.00, 128.58, 128.51, 128.27, 127.69, 127.28, 126.43, 126.16, 125.81, 124.47, 121.36, 118.54, 107.58, 77.48, 77.16, 76.84. MS(ESI) *m/z*: 424.1 $[M]^+$.

(*Z*)-3-benzylidene-5-nitro-2-(quinolin-8-yl)isoindolin-1-one(3h)

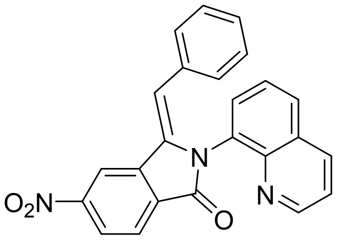

^{1}H NMR (400 MHz, $CDCl_3$) δ 8.83 (dd, J = 4.2, 1.7 Hz, 1H), 8.76 (d, J = 1.8 Hz, 1H), 8.42 (dd, J = 8.3, 1.9 Hz, 1H), 8.15 (d, J = 8.3 Hz, 1H), 8.00 (dd, J = 8.3, 1.7 Hz, 1H), 7.63 (dd, J = 8.2, 1.3 Hz, 1H), 7.51 (dd, J = 7.4, 1.4 Hz, 1H), 7.36 – 7.31 (m, 2H), 6.97 (s, 1H), 6.76 – 6.70 (m, 1H), 6.56 (d, J = 4.7 Hz, 4H). ^{13}C NMR (101 MHz, $CDCl_3$) δ 166.11, 150.86, 150.64, 144.02, 139.58, 136.09, 134.65, 133.49, 132.79, 132.59, 130.03, 129.03, 128.17, 126.81, 126.59, 125.82, 125.28, 124.09, 121.61, 115.70, 110.61, 77.48, 77.16, 76.84. MS(ESI) *m/z*: 393.1 $[M]^+$.

(*Z*)-3-(4-chlorobenzylidene)-2-(quinolin-8-yl)isoindolin-1-one(3i)

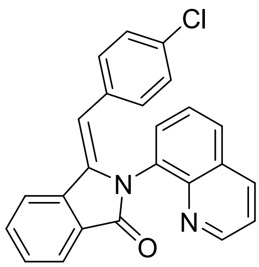

^{1}H NMR (400 MHz, $CDCl_3$) δ 8.83 (dd, J = 4.2, 1.7 Hz, 1H), 8.04 – 7.97 (m, 2H), 7.87 (d, J = 7.8 Hz, 1H), 7.69 (dd, J = 13.6, 7.3 Hz, 2H), 7.58 (t, J = 7.5 Hz, 1H), 7.51 (d, J = 7.3

Hz, 1H), 7.38 – 7.30 (m, 2H), 6.71 (s, 1H), 6.50 – 6.43 (m, 4H). ^{13}C NMR (101 MHz, $CDCl_3$) δ 168.13, 150.54, 144.38, 138.52, 136.86, 136.01, 134.17, 132.49, 132.09, 131.91, 130.25, 129.43, 129.38, 129.00, 128.62, 128.39, 126.36, 125.89, 124.14, 121.54, 119.81, 105.91, 77.48, 77.16, 76.84. MS(ESI) *m/z*: 382.0 $[M]^+$.

(*Z*)-3-(2-chlorobenzylidene)-2-(quinolin-8-yl)isoindolin-1-one(3j)

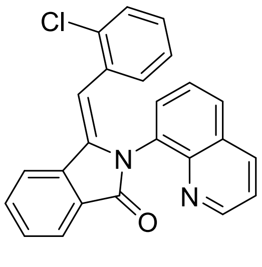

^{1}H NMR (400 MHz, $CDCl_3$) δ 8.89 (dd, *J* = 4.2, 1.6 Hz, 1H), 8.02 – 7.92 (m, 3H), 7.71 (t, *J* = 7.6 Hz, 1H), 7.62 – 7.54 (m, 3H), 7.34 – 7.28 (m, 2H), 6.86 (d, *J* = 8.0 Hz, 1H), 6.71 (s, 1H), 6.62 (t, *J* = 7.7 Hz, 1H), 6.35 (d, *J* = 7.7 Hz, 1H), 6.14 (t, *J* = 7.5 Hz, 1H). ^{13}C NMR (101 MHz, $CDCl_3$) δ 168.24, 150.79, 144.49, 138.40, 137.24, 135.85, 133.82, 133.01, 132.50, 132.44, 130.49, 130.00, 129.52, 128.76, 128.62, 128.54, 127.89, 127.72, 125.77, 124.34, 124.09, 121.39, 120.13, 104.37, 77.48, 77.16, 76.84. MS(ESI) *m/z*: 382.0 $[M]^+$.

(*Z*)-3-(2-iodobenzylidene)-2-(quinolin-8-yl)isoindolin-1-one(3k)

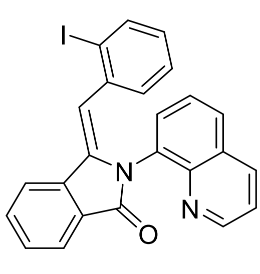

^{1}H NMR (400 MHz, $CDCl_3$) δ 8.94 (dd, *J* = 3.3, 0.8 Hz, 1H), 8.02 – 7.97 (m, 2H), 7.95 (d, *J* = 7.8 Hz, 1H), 7.71 (t, *J* = 7.6 Hz, 1H), 7.60 (d, *J* = 8.1 Hz, 1H), 7.58 – 7.55 (m, 1H), 7.54 (s, 1H), 7.35 (d, *J* = 6.8 Hz, 1H), 7.33 – 7.26 (m, 2H), 6.58 (s, 1H), 6.39 – 6.33 (m, 2H), 6.21 (t, *J* = 7.5 Hz, 1H). ^{13}C NMR (101 MHz, $CDCl_3$) δ 168.23, 150.99, 144.46, 138.38, 137.86, 137.25, 136.58, 135.93, 133.80, 132.51, 130.14, 129.87, 129.50, 128.76, 128.64, 128.48, 127.81, 125.79, 125.75, 124.12, 121.42, 120.03, 110.49, 99.83, 77.48, 77.16, 76.84. MS(ESI) *m/z*: 474.0 $[M]^+$.

(*Z*)-3-(2-methoxybenzylidene)-2-(quinolin-8-yl)isoindolin-1-one(3l)

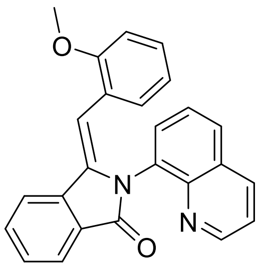

^{1}H NMR (400 MHz, $CDCl_3$) δ 8.86 (dd, *J* = 4.2, 1.7 Hz, 1H), 7.99 – 7.98 (m, 1H), 7.97 – 7.95 (m, 1H), 7.92 (d, *J* = 7.8 Hz, 1H), 7.67 (td, *J* = 7.6, 1.1 Hz, 1H), 7.55 (td, *J* = 7.9, 4.1 Hz, 2H), 7.48 (dd, *J* = 7.3, 1.4 Hz, 1H), 7.31 – 7.27 (m, 2H), 6.76 (s, 1H), 6.67 (ddd, *J* = 8.5, 7.6, 1.2 Hz, 1H), 6.30 (t, *J* = 8.2 Hz, 2H), 5.92 (td, *J* = 7.4, 0.7 Hz, 1H), 3.58 (s, 3H). ^{13}C NMR (101 MHz, $CDCl_3$) δ 168.33, 156.18, 150.34, 144.60, 138.77, 136.38, 135.95, 134.10, 132.22, 130.05, 130.00, 129.00, 128.79, 128.46, 128.39, 127.93, 125.52, 123.91, 122.52, 121.11, 120.10, 118.57, 108.84, 103.92, 77.48, 77.16, 76.84, 55.03. MS(ESI) *m/z*: 378.1 $[M]^{+}$.

(*Z*)-3-(4-methylbenzylidene)-2-(quinolin-8-yl)isoindolin-1-one(3n)

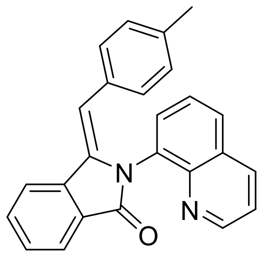

^{1}H NMR (400 MHz, $CDCl_3$) δ 8.83 (dd, *J* = 4.1, 1.5 Hz, 1H), 7.97 (t, *J* = 7.8 Hz, 2H), 7.85 (d, *J* = 7.8 Hz, 1H), 7.65 (t, *J* = 7.6 Hz, 1H), 7.59 – 7.51 (m, 2H), 7.45 (d, *J* = 7.4 Hz, 1H), 7.30 – 7.25 (m, 2H), 6.78 (s, 1H), 6.42 (d, *J* = 7.8 Hz, 2H), 6.31 (d, *J* = 7.8 Hz, 2H), 1.99 (s, 3H). ^{13}C NMR (101 MHz, $CDCl_3$) δ 168.17, 150.38, 144.52, 138.81, 135.82, 135.72, 134.38, 132.25, 130.53, 130.07, 128.99, 128.91, 128.28, 128.14, 128.06, 127.00, 125.72, 123.93, 121.29, 119.67, 107.67, 77.48, 77.16, 76.84, 20.95. MS(ESI) *m/z*:

362.1 [M]$^+$.

(*Z*)-3-(4-propylbenzylidene)-2-(quinolin-8-yl)isoindolin-1-one(3o)

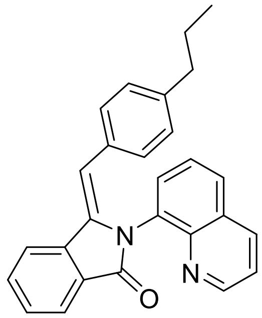

^{1}H NMR (400 MHz, CDCl$_3$) δ 8.83 (dd, *J* = 4.2, 1.7 Hz, 1H), 8.00 – 7.92 (m, 2H), 7.86 (d, *J* = 7.8 Hz, 1H), 7.66 (td, *J* = 7.6, 1.1 Hz, 1H), 7.54 (dd, *J* = 7.8, 6.9 Hz, 2H), 7.47 (dd, *J* = 7.4, 1.3 Hz, 1H), 7.30 – 7.26 (m, 2H), 6.79 (s, 1H), 6.45 (d, *J* = 7.9 Hz, 2H), 6.32 (d, *J* = 8.0 Hz, 2H), 2.22 (t, *J* = 7.5 Hz, 2H), 1.42 – 1.32 (m, 2H), 0.80 (t, *J* = 7.3 Hz, 3H). ^{13}C NMR (101 MHz, CDCl$_3$) δ 168.18, 150.40, 144.54, 140.56, 138.80, 135.89, 135.81, 134.38, 132.26, 130.89, 130.11, 129.02, 128.88, 128.32, 128.08, 126.53, 125.72, 123.98, 121.25, 119.70, 107.73, 77.48, 77.16, 76.84, 37.53, 24.47, 13.80. MS(ESI) *m/z*: 390.1 [M]$^+$.

(*Z*)-4-benzylidene-5-(quinolin-8-yl)-4,5-dihydro-6H-thieno[2,3-c]pyrrol-6-one(3p)

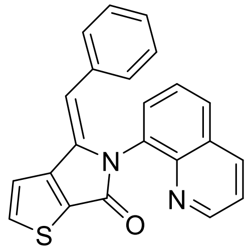

^{1}H NMR (400 MHz, CDCl$_3$) δ 8.92 (dd, *J* = 4.2, 1.7 Hz, 1H), 8.08 (dd, *J* = 8.3, 1.7 Hz, 1H), 7.76 (d, *J* = 5.2 Hz, 1H), 7.72 (dd, *J* = 8.2, 1.3 Hz, 1H), 7.51 (dd, *J* = 7.3, 1.4 Hz, 1H), 7.44 – 7.39 (m, 1H), 7.37 (dd, *J* = 8.3, 4.2 Hz, 1H), 7.29 (d, *J* = 5.2 Hz, 1H), 7.09 (d, *J* = 6.9 Hz, 2H), 7.04 – 6.99 (m, 1H), 6.94 (t, *J* = 7.4 Hz, 2H), 6.79 (s, 1H). ^{13}C NMR (101 MHz, CDCl$_3$) δ 159.32, 151.10, 146.16, 145.52, 144.79, 136.93, 136.37, 136.27, 134.06, 130.99, 129.48, 129.00, 128.96, 128.87, 128.12, 127.41, 125.89, 124.66, 121.68, 104.66, 77.48, 77.16, 76.84. MS(ESI) *m/z*: 354.0 [M]$^+$.

(*Z*)-3-(cyclohexylmethylene)-2-(quinolin-8-yl)isoindolin-1-one(3q)

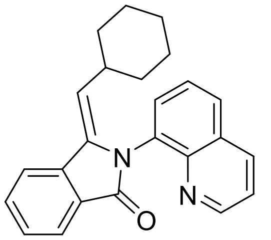

^{1}H NMR (400 MHz, $CDCl_3$) δ 8.89 (dd, *J* = 4.2, 1.7 Hz, 1H), 8.24 (dd, *J* = 8.3, 1.7 Hz, 1H), 8.00 – 7.92 (m, 2H), 7.80 (dd, *J* = 7.3, 1.4 Hz, 1H), 7.72 (d, *J* = 7.8 Hz, 1H), 7.69 – 7.64 (m, 1H), 7.60 (td, *J* = 7.6, 1.1 Hz, 1H), 7.48 (td, *J* = 7.5, 0.8 Hz, 1H), 7.42 (dd, *J* = 8.3, 4.2 Hz, 1H), 5.45 (d, *J* = 10.7 Hz, 1H), 1.72 (dt, *J* = 14.6, 6.7 Hz, 1H), 1.48 – 1.42 (m, 1H), 1.41 – 1.35 (m, 1H), 1.26 (d, *J* = 7.0 Hz, 2H), 1.23 – 1.17 (m, 1H), 1.15 – 1.07 (m, 1H), 0.93 – 0.85 (m, 2H), 0.74 (ddd, *J* = 12.0, 10.8, 3.5 Hz, 2H). ^{13}C NMR (101 MHz, $CDCl_3$) δ 168.48, 151.33, 145.68, 138.84, 136.21, 135.72, 134.04, 131.93, 130.67, 129.30, 129.27, 128.42, 128.39, 126.35, 123.76, 121.92, 119.40, 115.12, 77.48, 77.16, 76.84, 35.28, 33.32, 33.15, 25.69, 25.57, 25.45. MS(ESI) *m/z*: 353.1 $[M]^+$.

(*Z*)-3-(cyclopropylmethylene)-2-(quinolin-8-yl)isoindolin-1-one(3r)

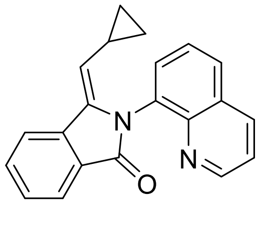

^{1}H NMR (400 MHz, $CDCl_3$) δ 8.92 (dd, *J* = 4.2, 1.7 Hz, 1H), 8.19 (dd, *J* = 8.3, 1.7 Hz, 1H), 7.93 (d, *J* = 7.6 Hz, 1H), 7.90 (dd, *J* = 8.2, 1.3 Hz, 1H), 7.85 (dd, *J* = 7.3, 1.3 Hz, 1H), 7.66 – 7.62 (m, 2H), 7.57 (td, *J* = 7.7, 0.9 Hz, 1H), 7.46 (t, *J* = 7.7 Hz, 1H), 7.41 (dd, *J* = 8.3, 4.2 Hz, 1H), 5.13 – 5.06 (m, 1H), 0.40 – 0.32 (m, 2H), 0.28 (dd, *J* = 9.2, 6.5 Hz, 1H), 0.11 (ddd, *J* = 11.9, 8.6, 3.6 Hz, 1H), -0.04 (tdd, *J* = 8.5, 6.4, 4.5 Hz, 1H). ^{13}C NMR (101 MHz, $CDCl_3$) δ 168.07, 151.23, 145.52, 138.15, 136.16, 135.46, 135.28, 131.89, 130.66, 129.20, 129.14, 128.16, 127.82, 126.26, 123.74, 121.82, 118.97, 113.93, 77.48, 77.16, 76.84, 8.76, 8.66. MS(ESI) *m/z*: 312.1 $[M]^+$.

5. ^{1}H NMR and ^{13}C NMR spectra of products 3

(*Z*)-3-benzylidene-2-(quinolin-8-yl)isoindolin-1-one(3a)

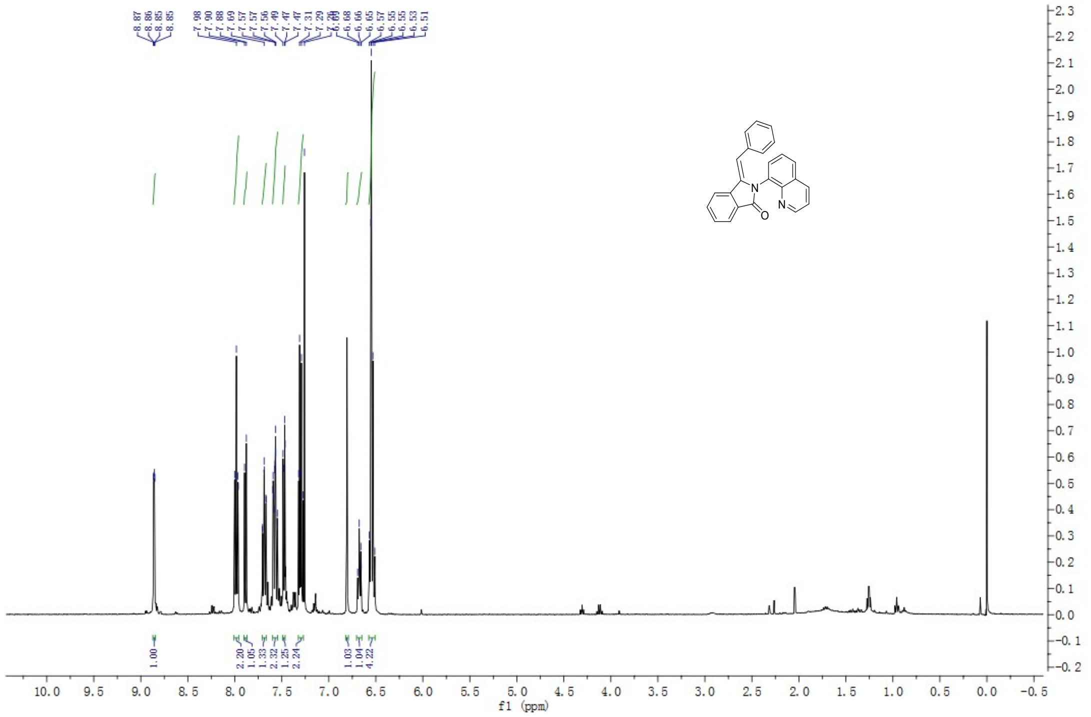

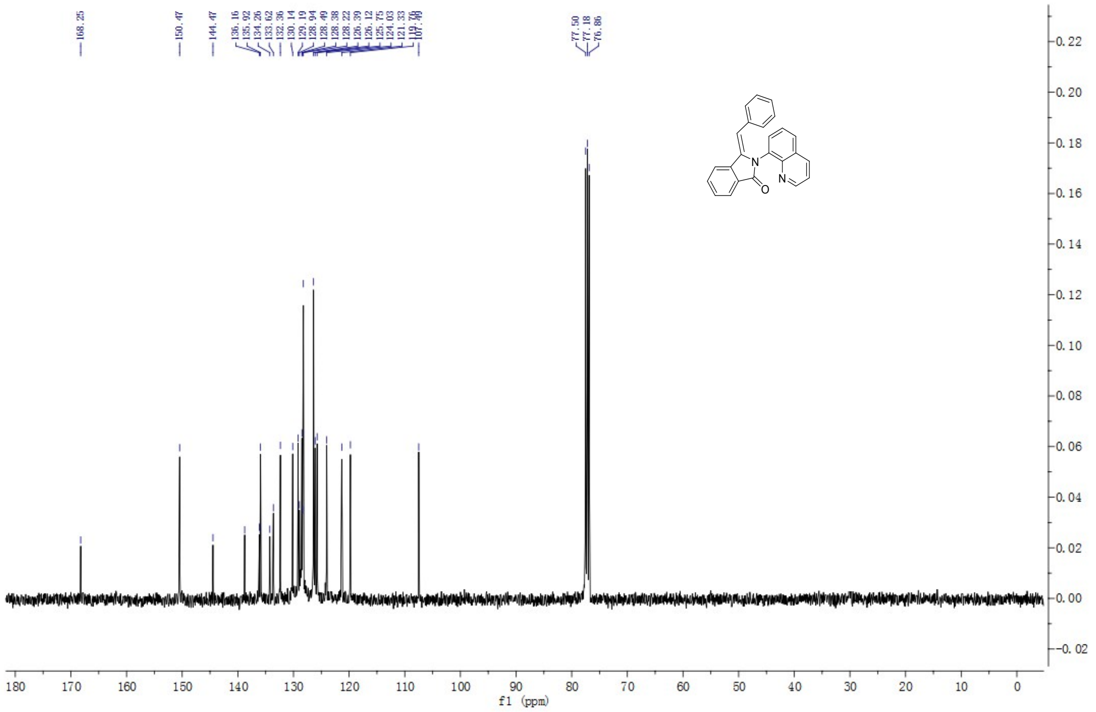

(*Z*)-3-benzylidene-5-fluoro-2-(quinolin-8-yl)isoindolin-1-one(3b)

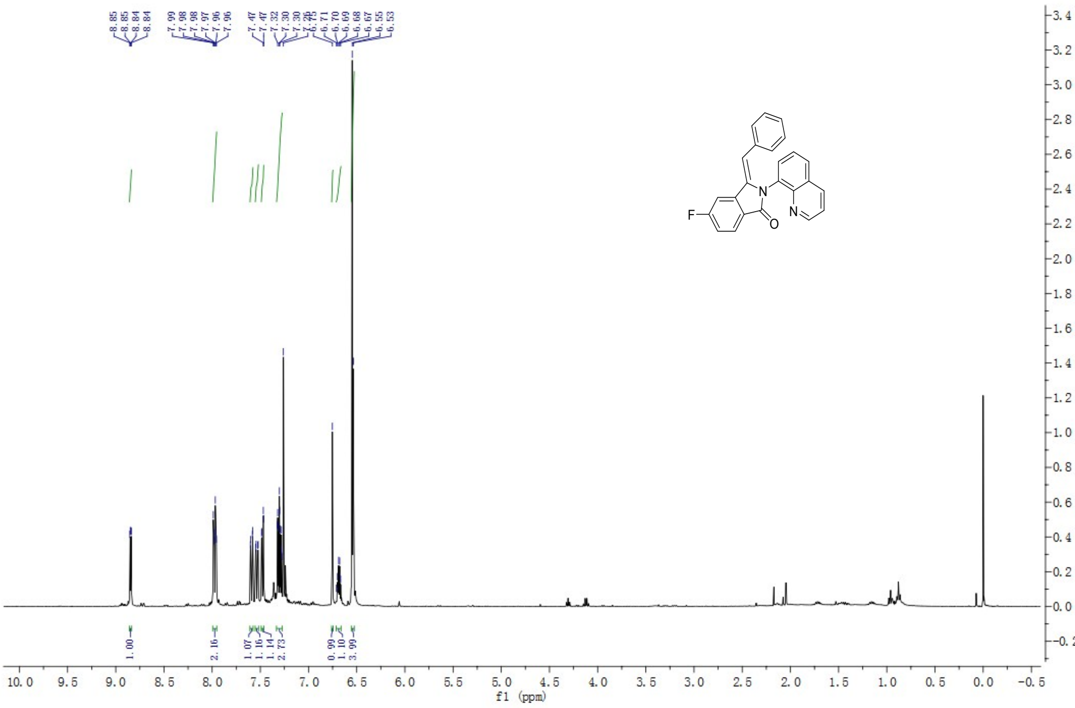

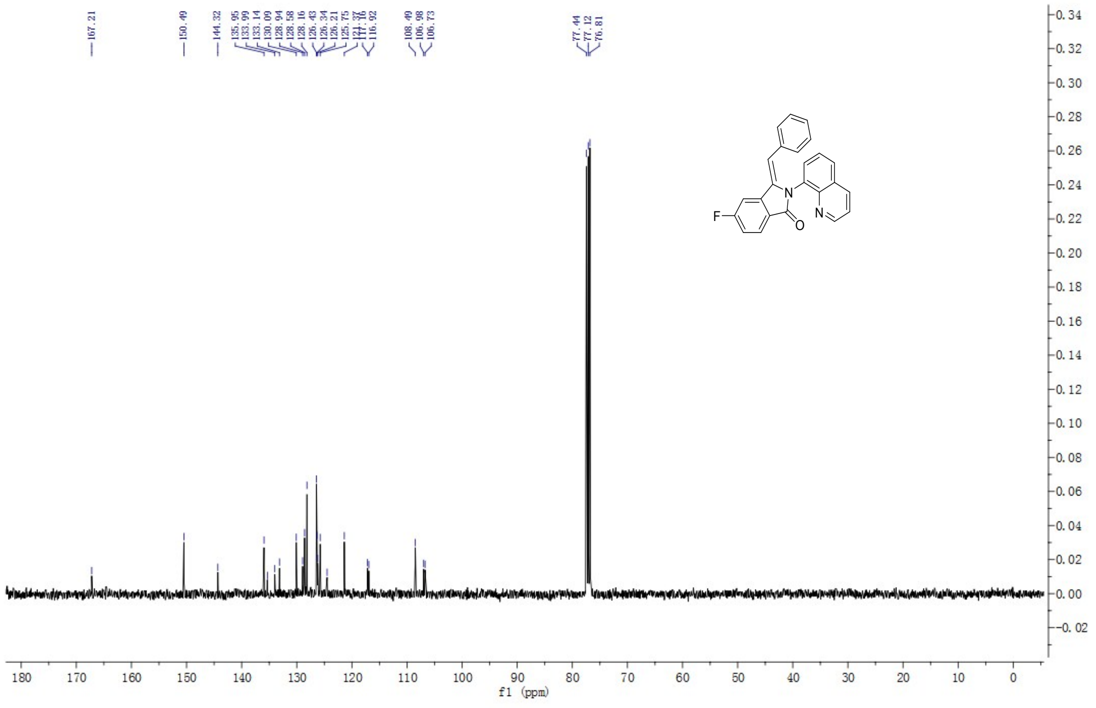

(*Z*)-3-benzylidene-5-chloro-2-(quinolin-8-yl)isoindolin-1-one(3c)

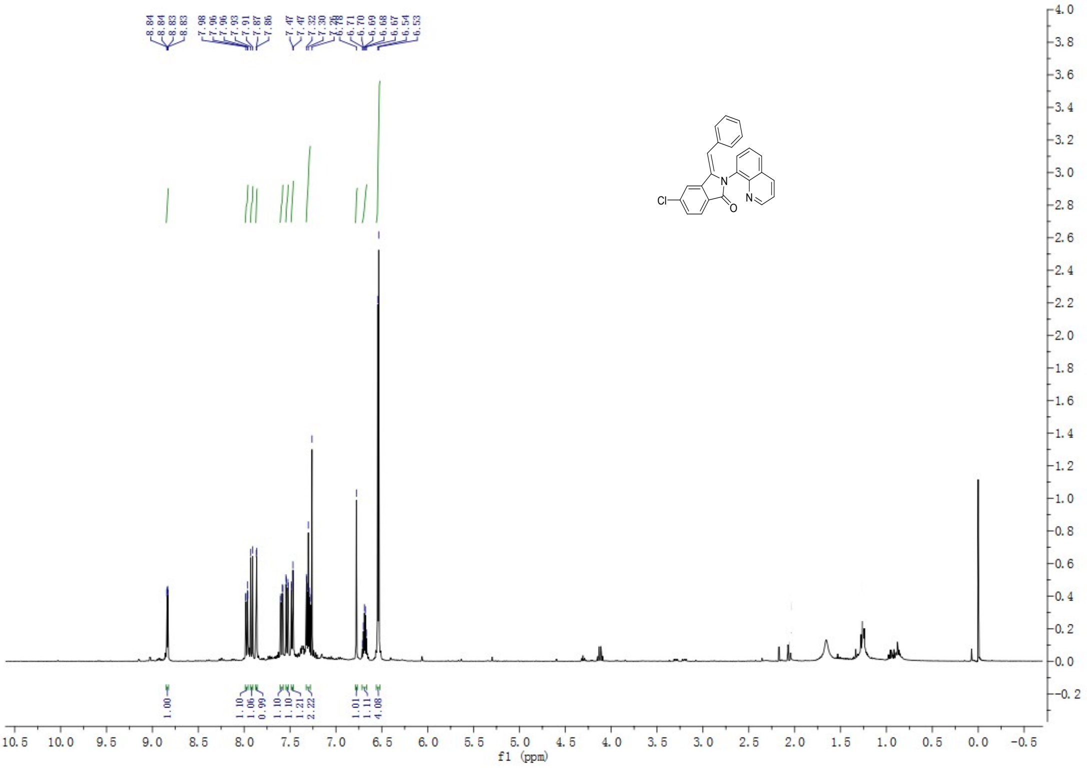

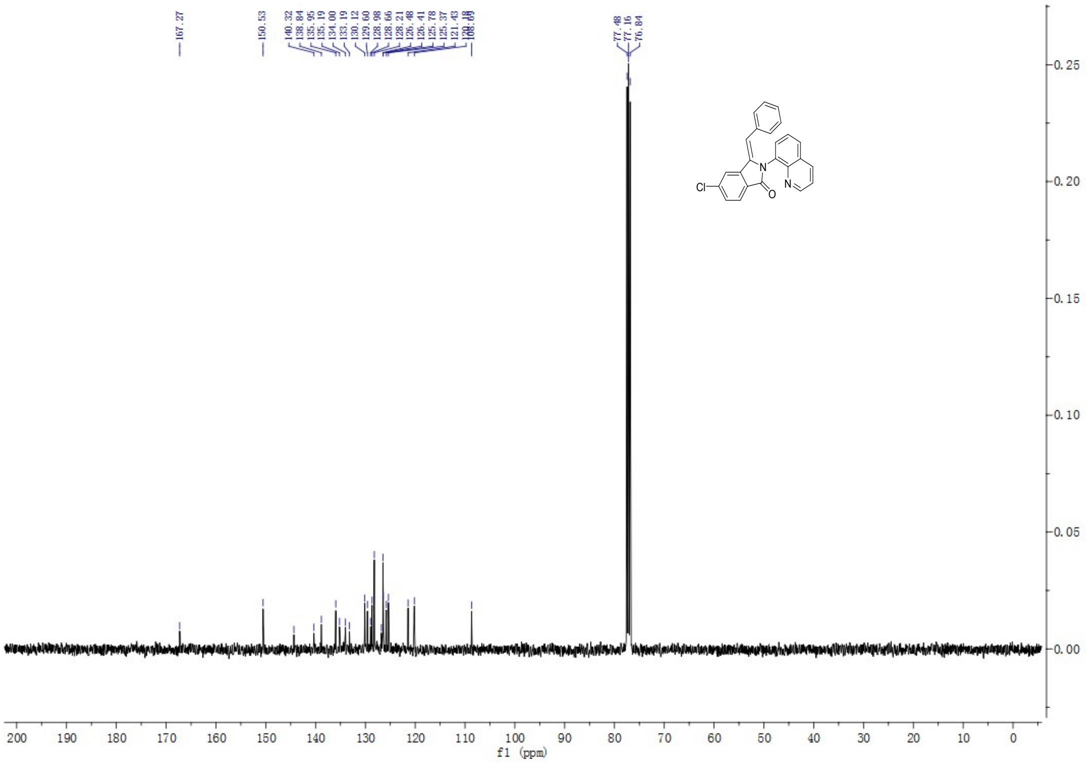

(*Z*)-3-benzylidene-5-methyl-2-(quinolin-8-yl)isoindolin-1-one(3d)

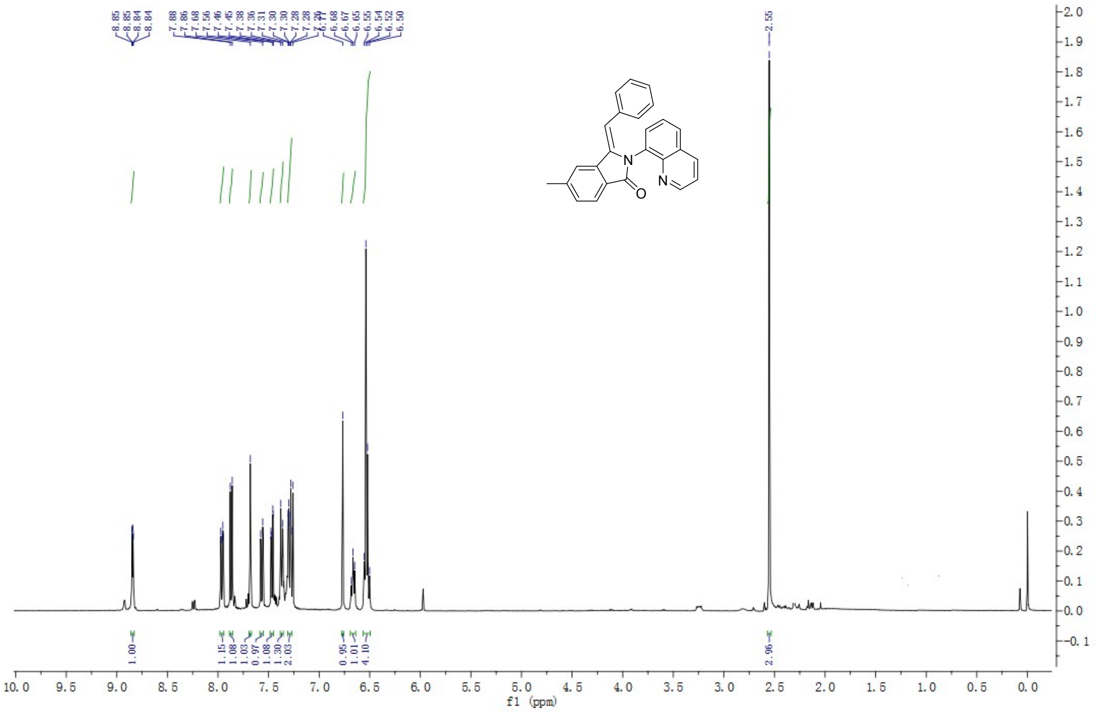

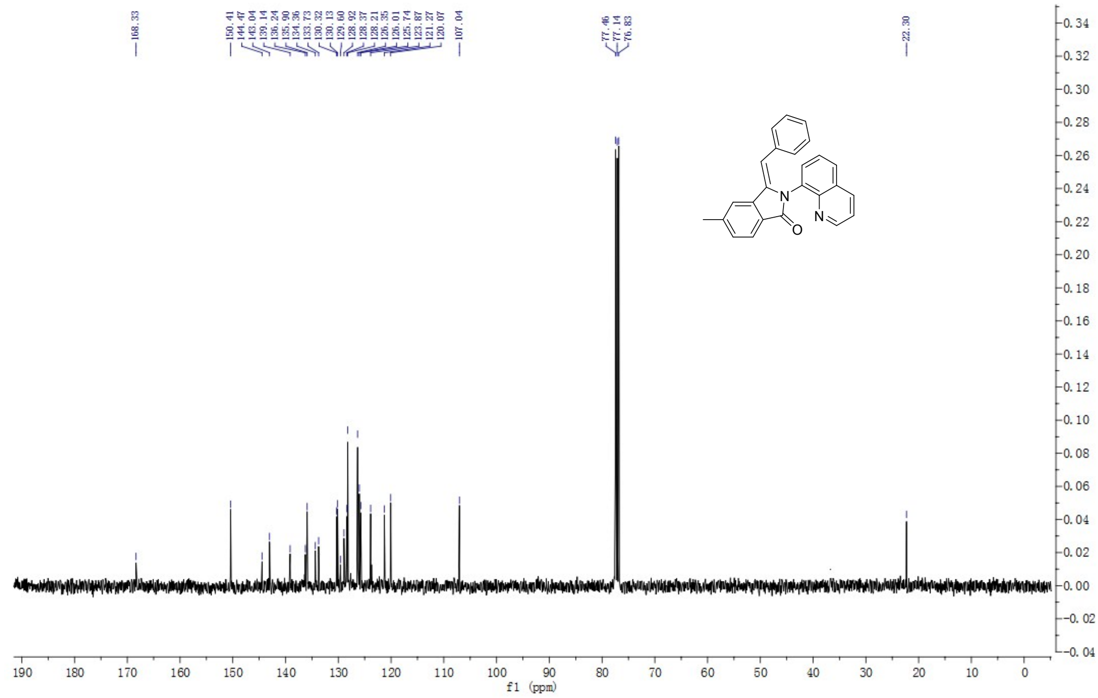

(*Z*)-3-benzylidene-5-methoxy-2-(quinolin-8-yl)isoindolin-1-one(3e)

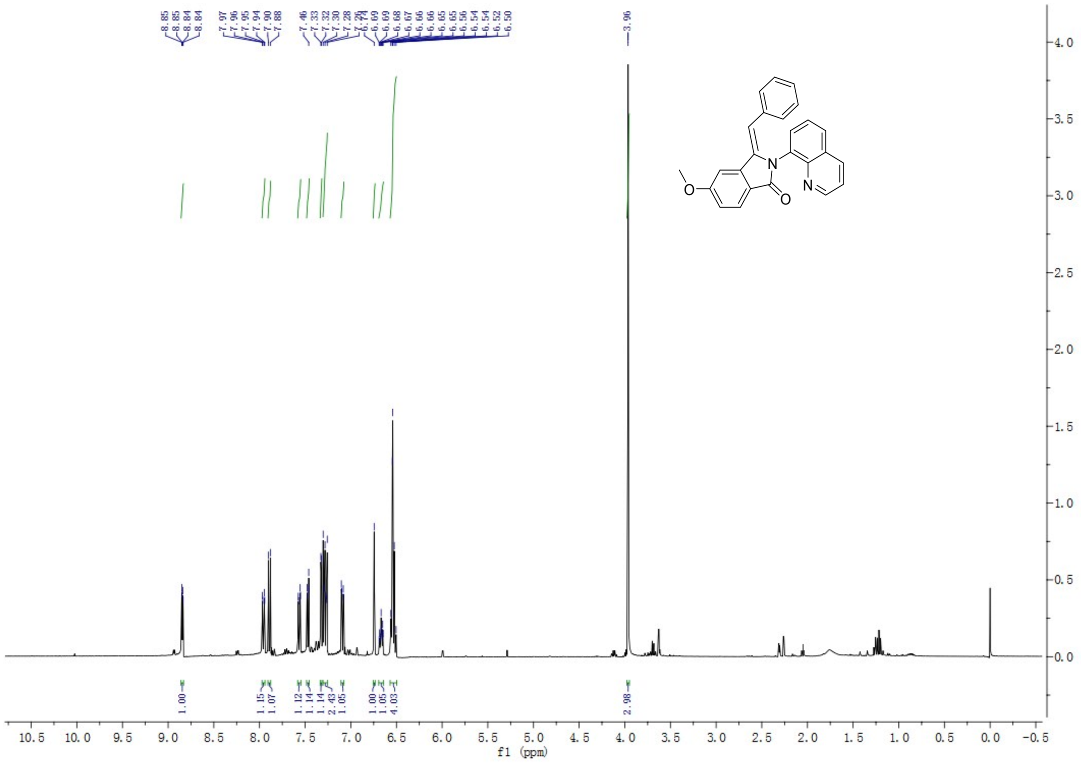

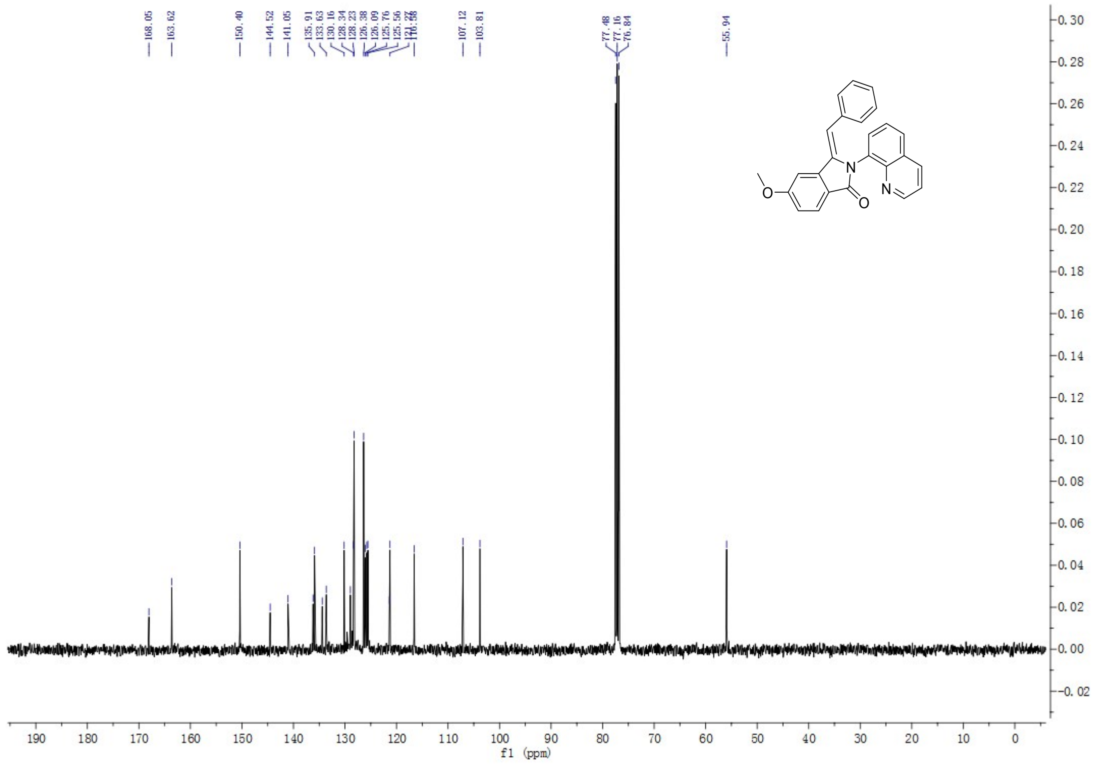

(*Z*)-3-benzylidene-6-methyl-2-(quinolin-8-yl)isoindolin-1-one(3f)

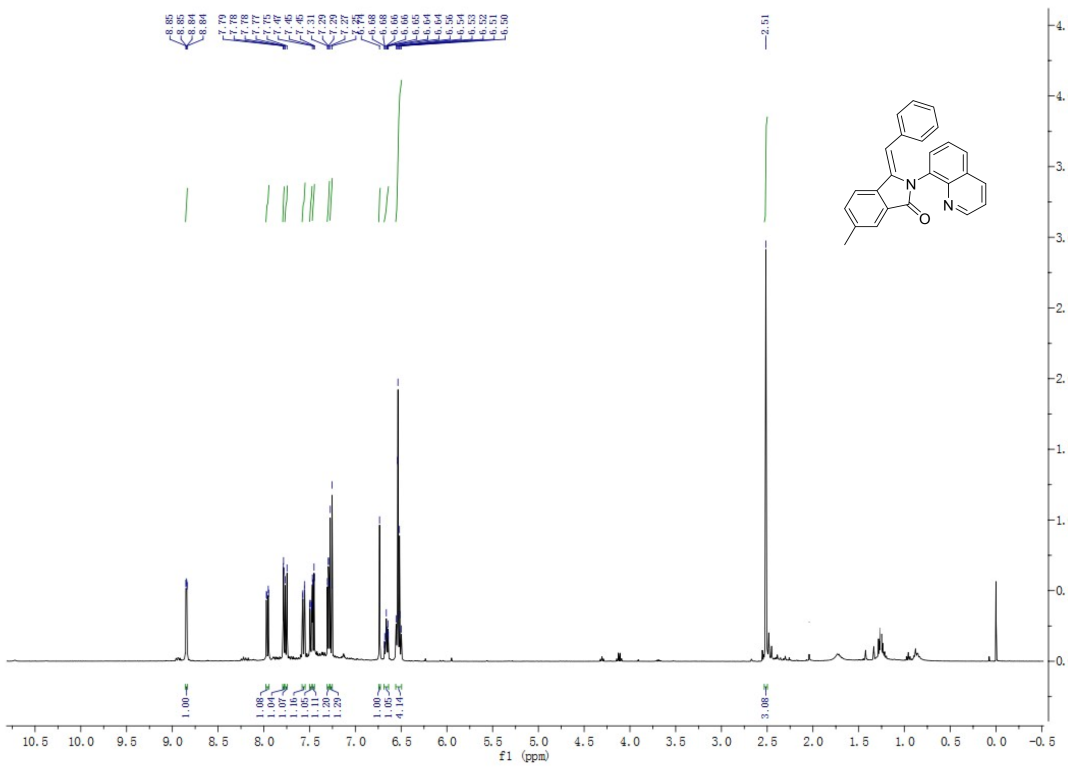

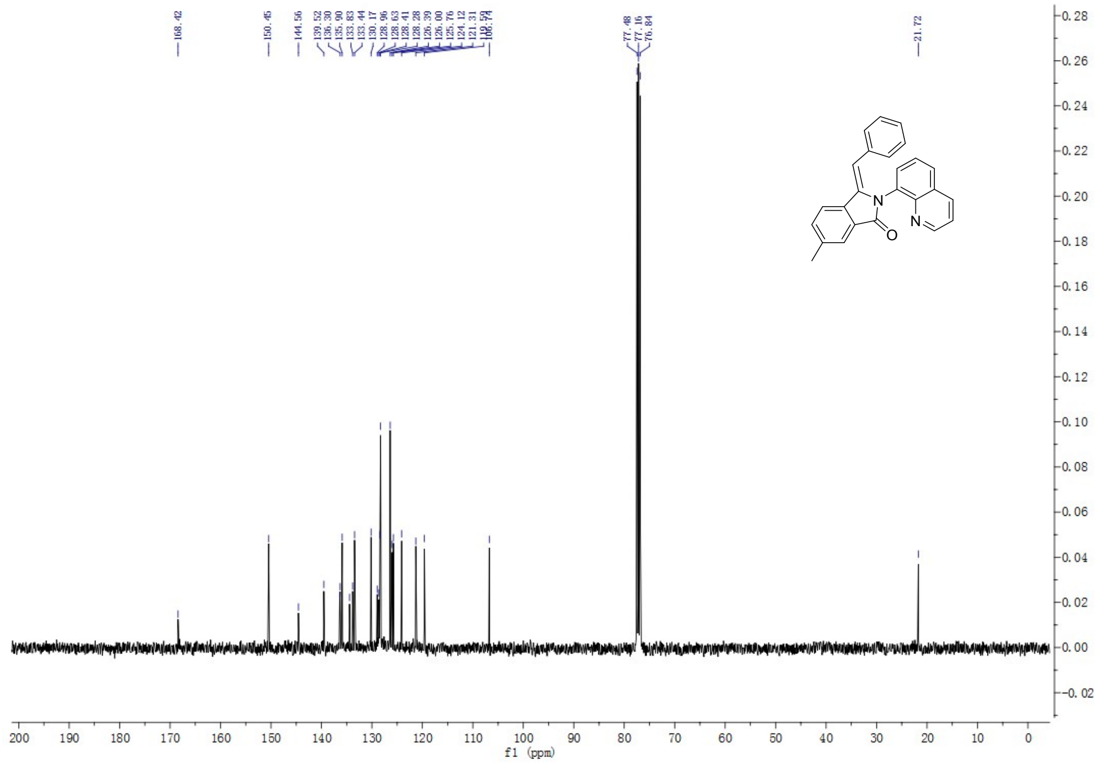

(*Z*)-3-benzylidene-5-phenyl-2-(quinolin-8-yl)isoindolin-1-one(3g)

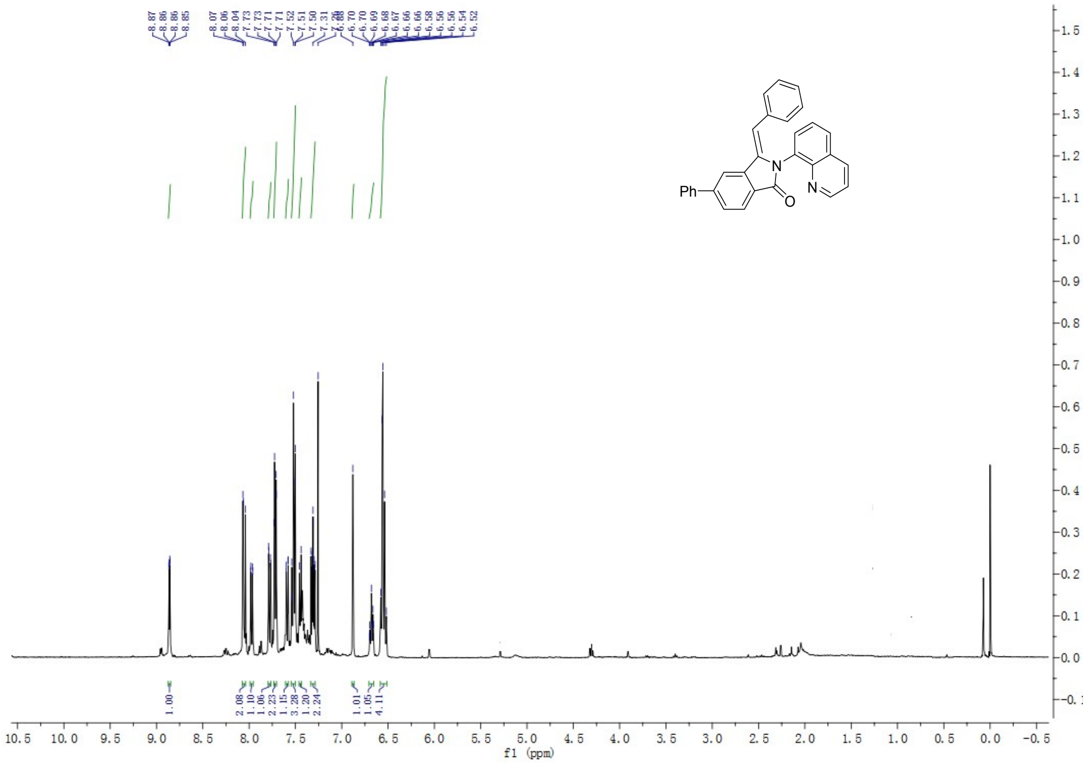

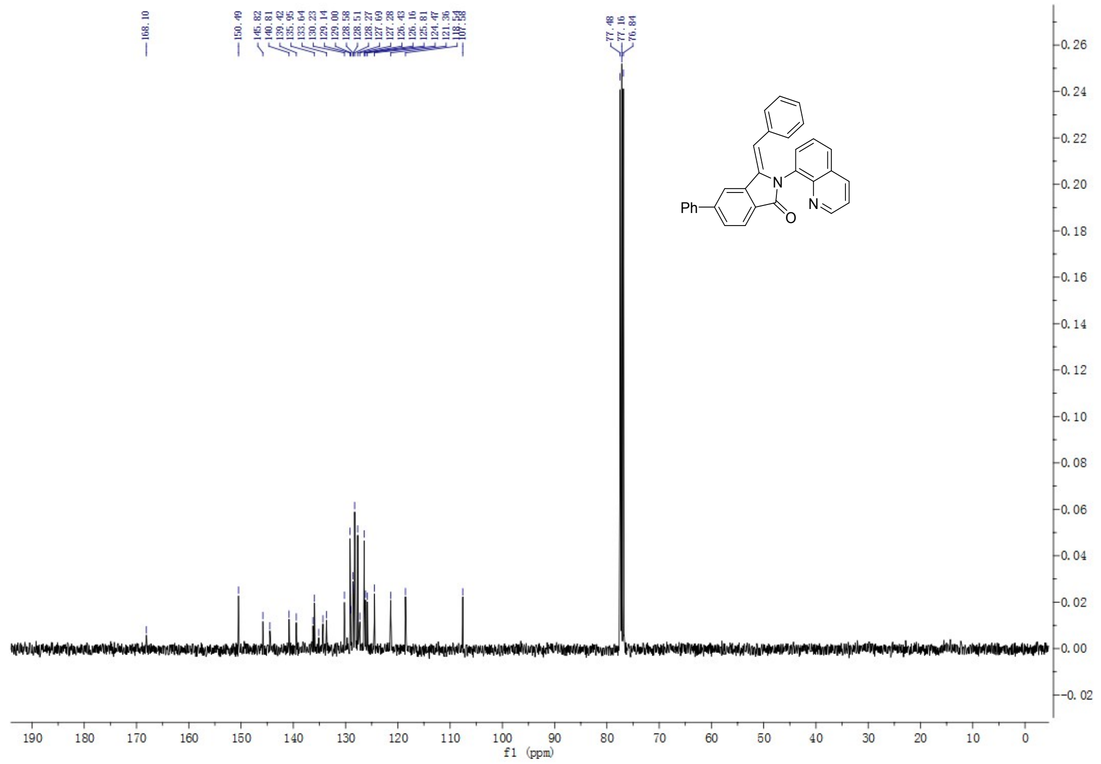

(*Z*)-3-benzylidene-5-nitro-2-(quinolin-8-yl)isoindolin-1-one(3h)

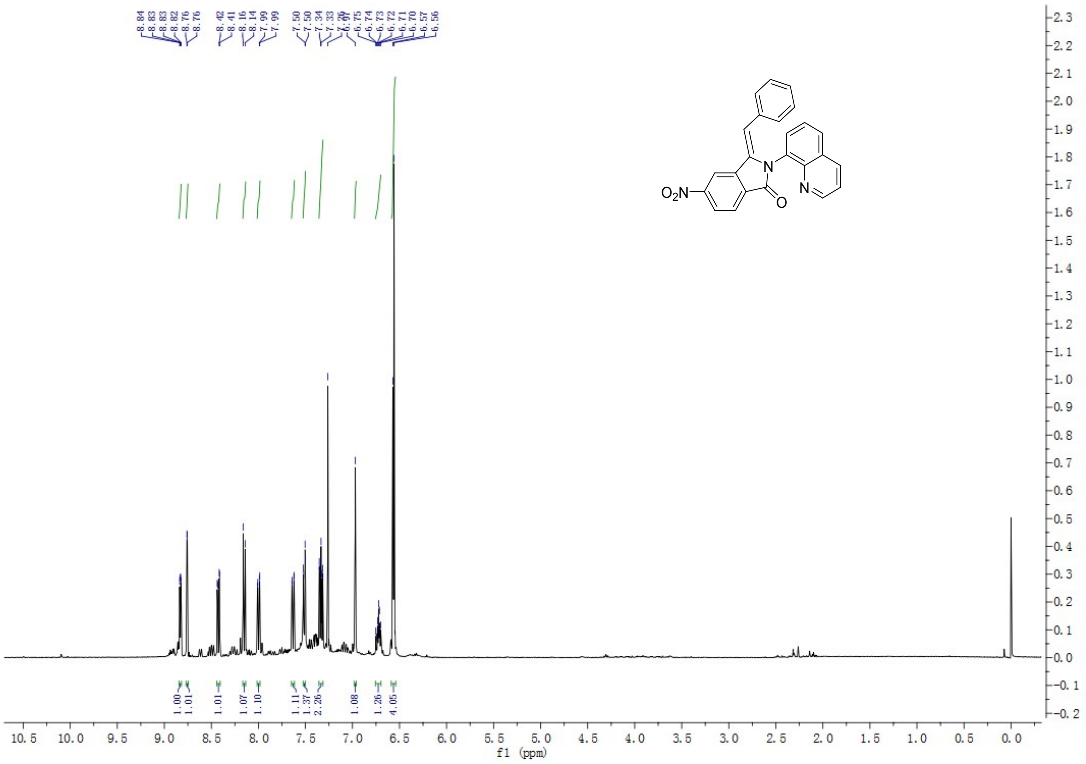

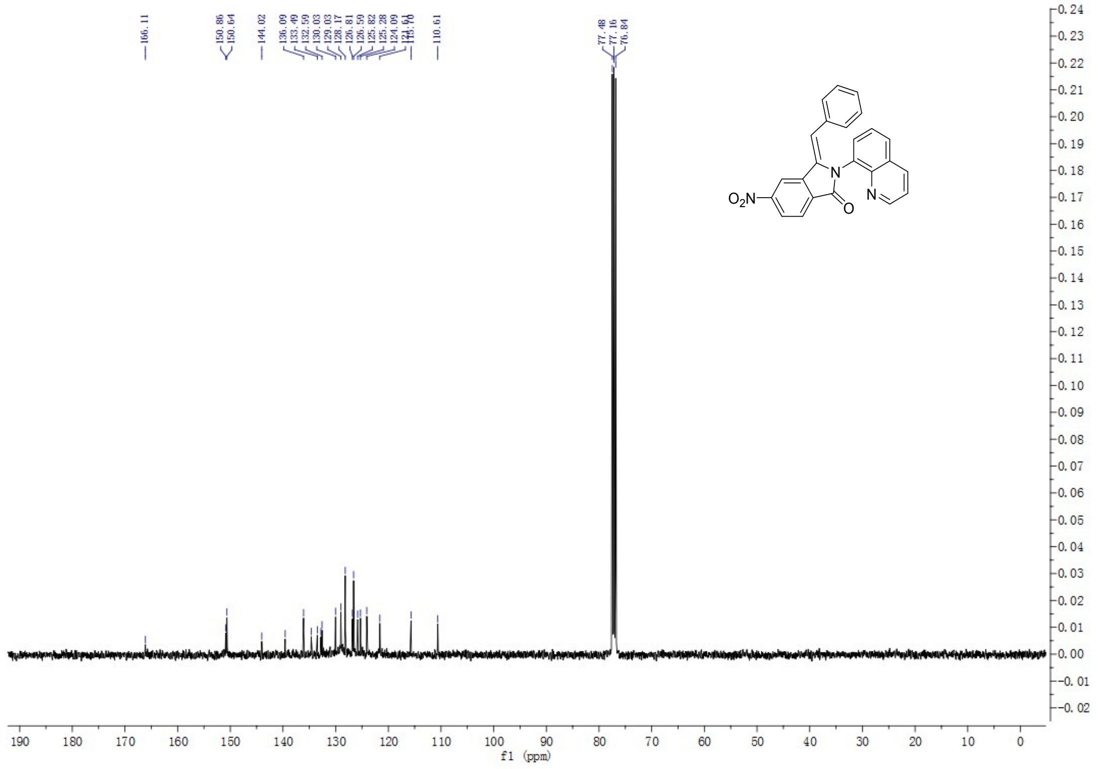

(*Z*)-3-(4-chlorobenzylidene)-2-(quinolin-8-yl)isoindolin-1-one(3i)

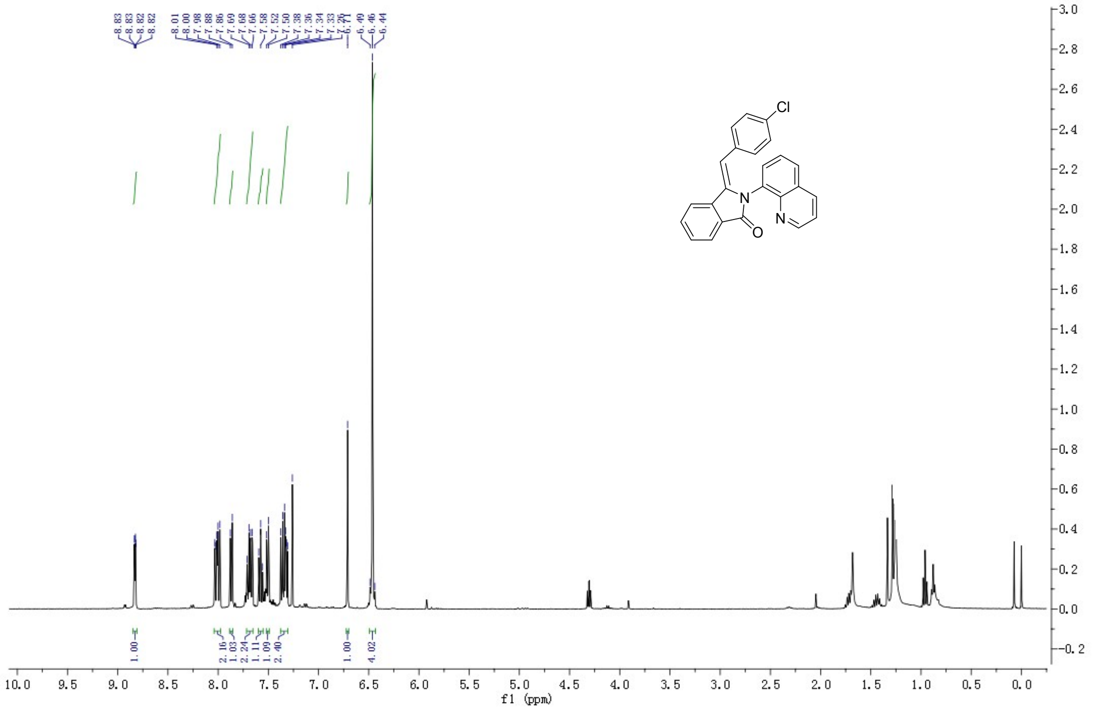

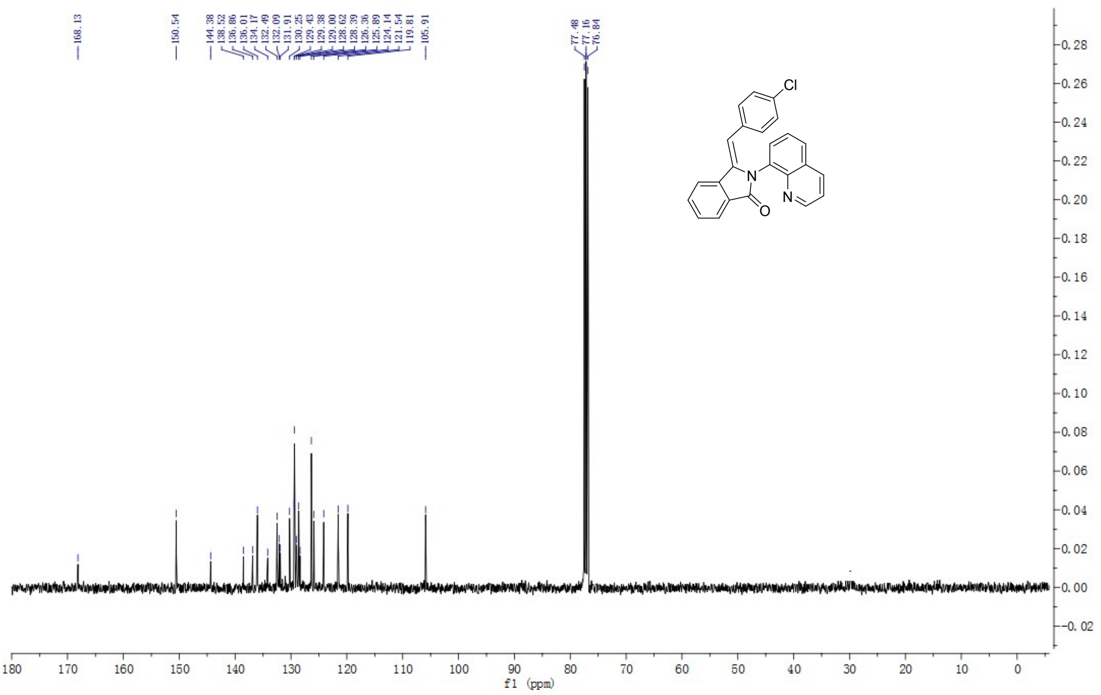

(*Z*)-3-(2-chlorobenzylidene)-2-(quinolin-8-yl)isoindolin-1-one(3j)

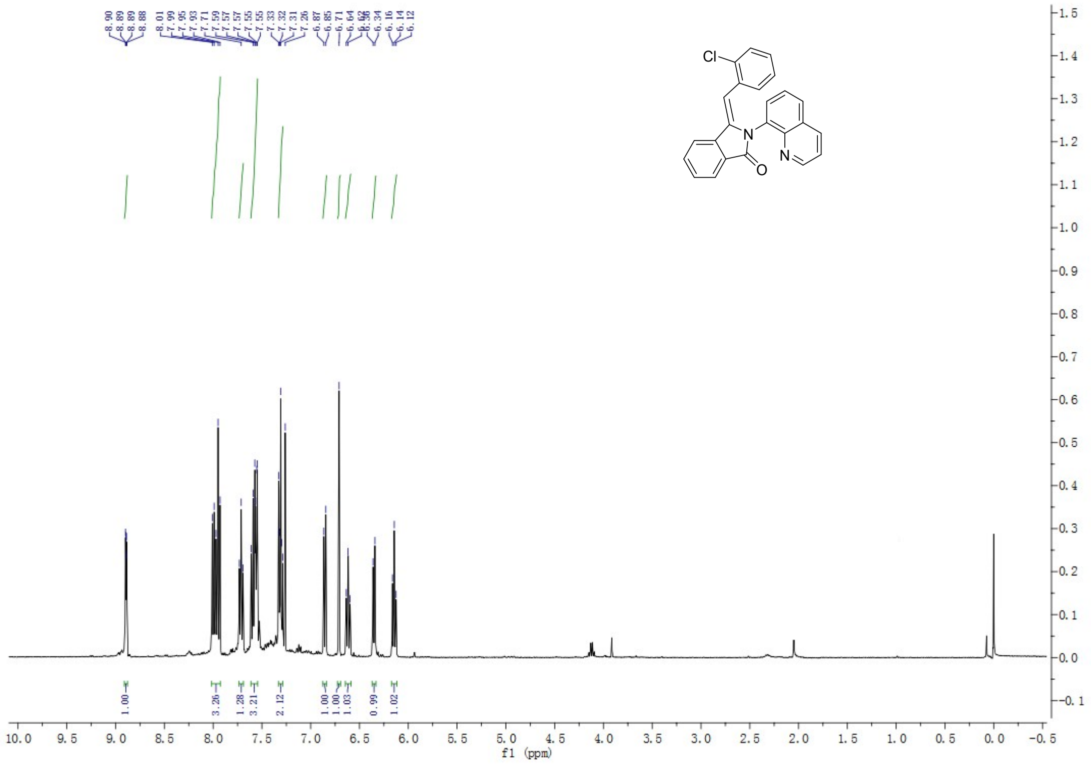

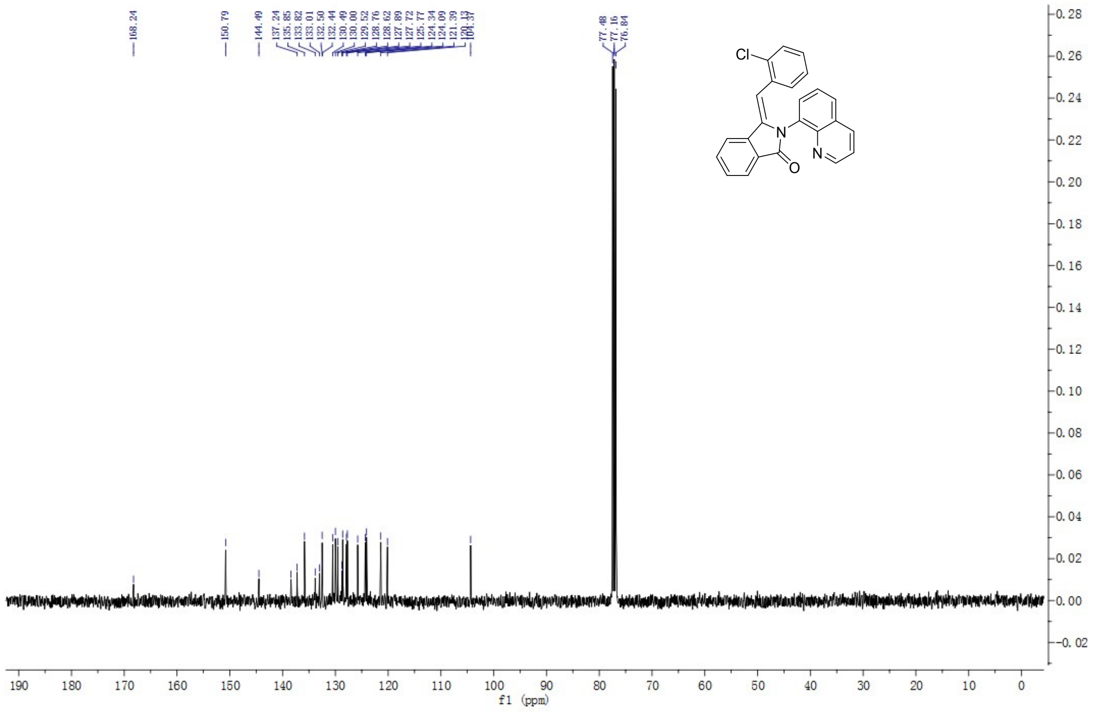

(*Z*)-3-(2-iodobenzylidene)-2-(quinolin-8-yl)isoindolin-1-one(3k)

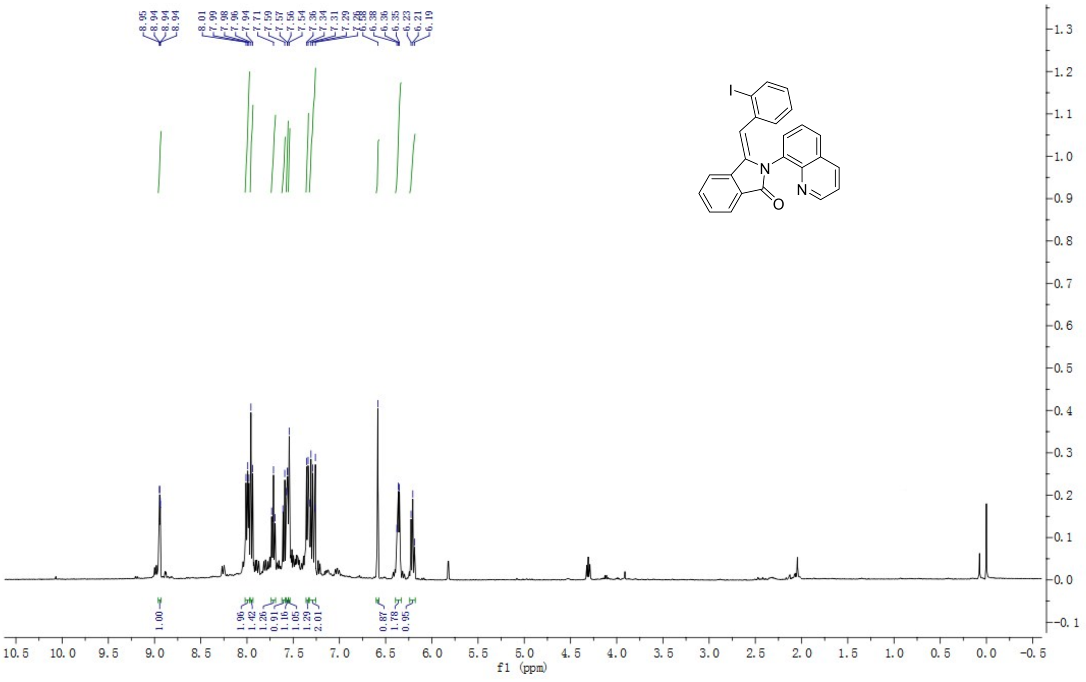

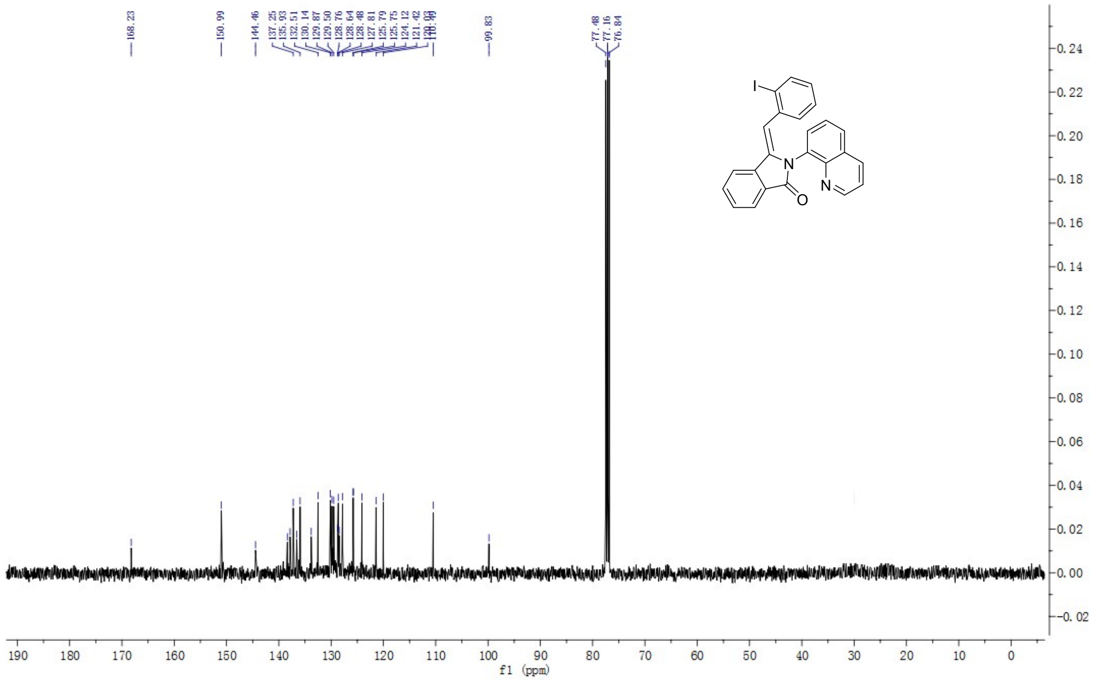

(*Z*)-3-(2-methoxybenzylidene)-2-(quinolin-8-yl)isoindolin-1-one(3l)

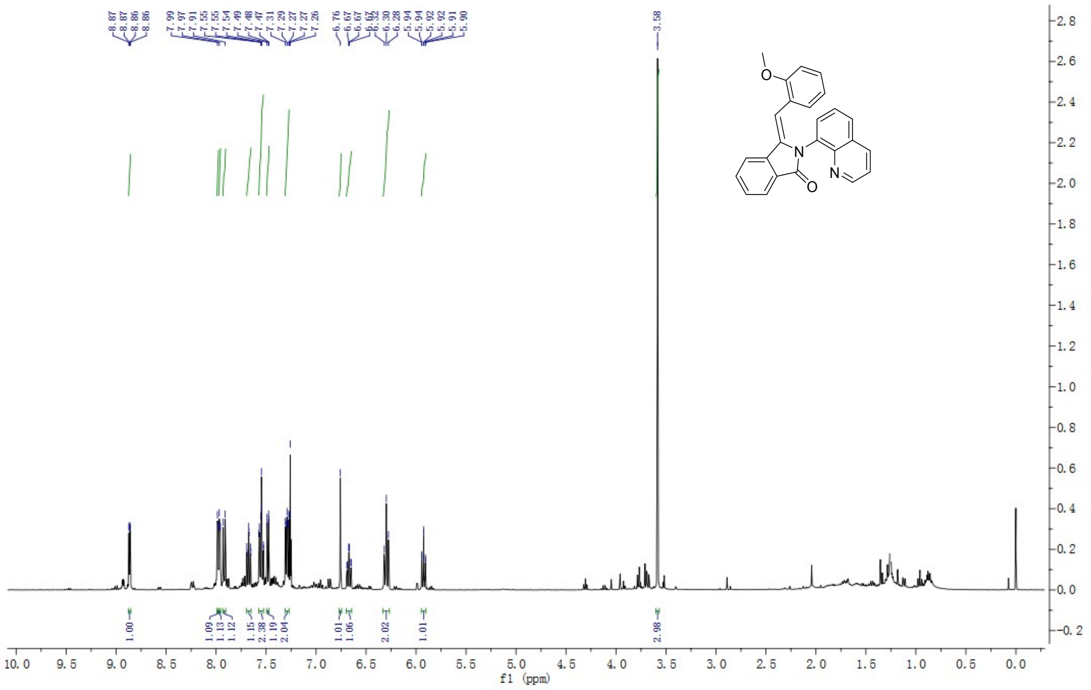

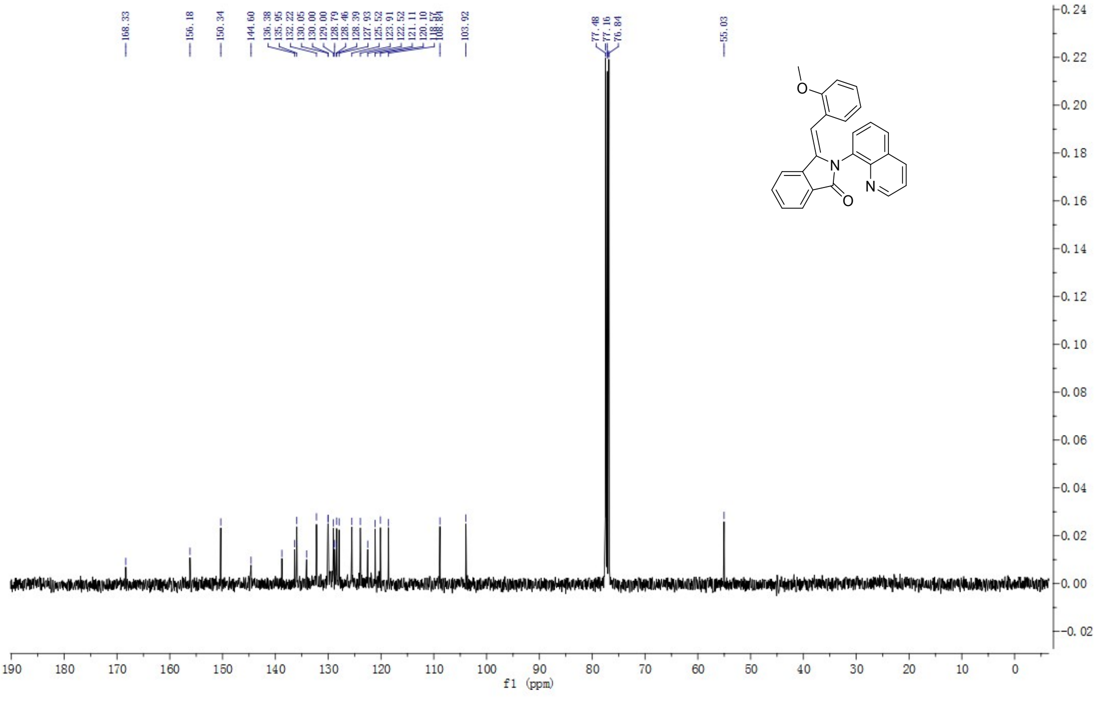

(*Z*)-3-(4-methylbenzylidene)-2-(quinolin-8-yl)isoindolin-1-one(3n)

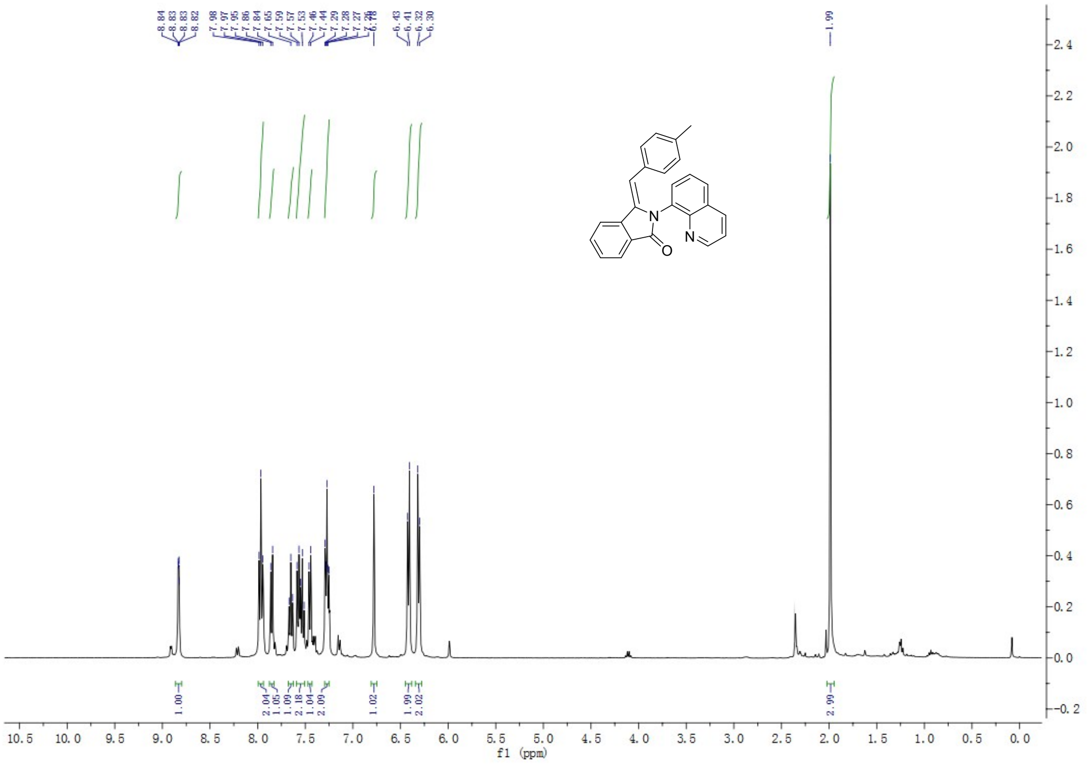

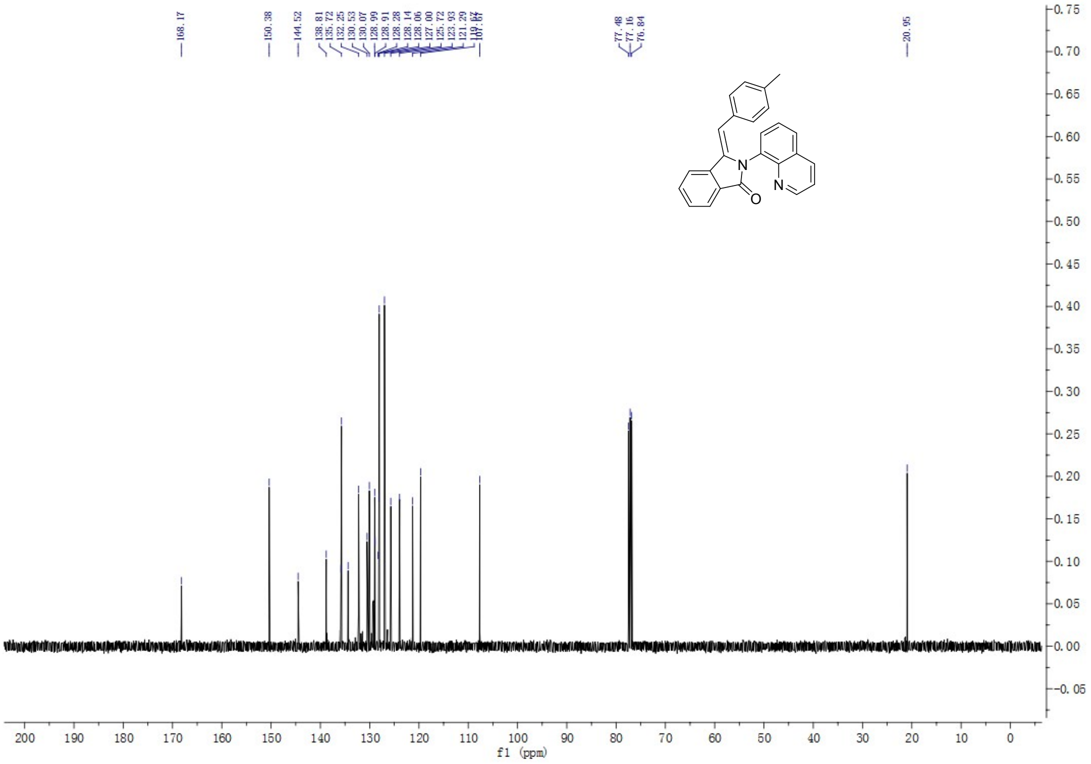

(*Z*)-3-(4-propylbenzylidene)-2-(quinolin-8-yl)isoindolin-1-one(3o)

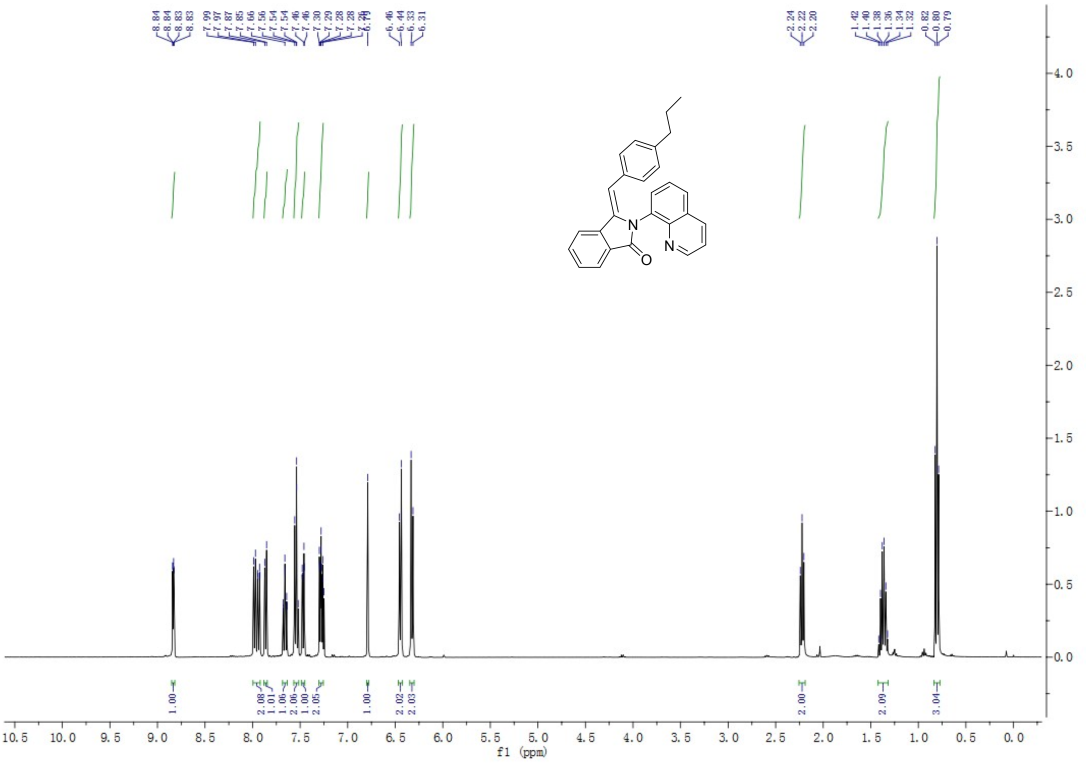

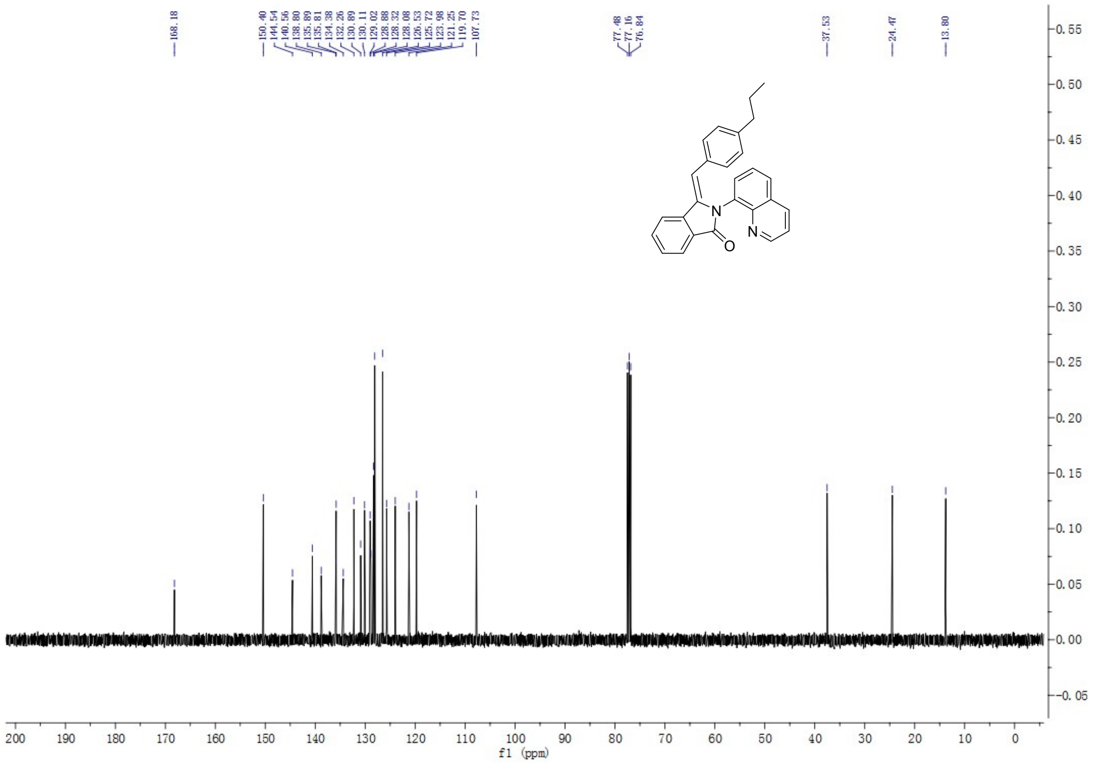

(*Z*)-4-benzylidene-5-(quinolin-8-yl)-4,5-dihydro-6H-thieno[2,3-c]pyrrol-6-one(3p)

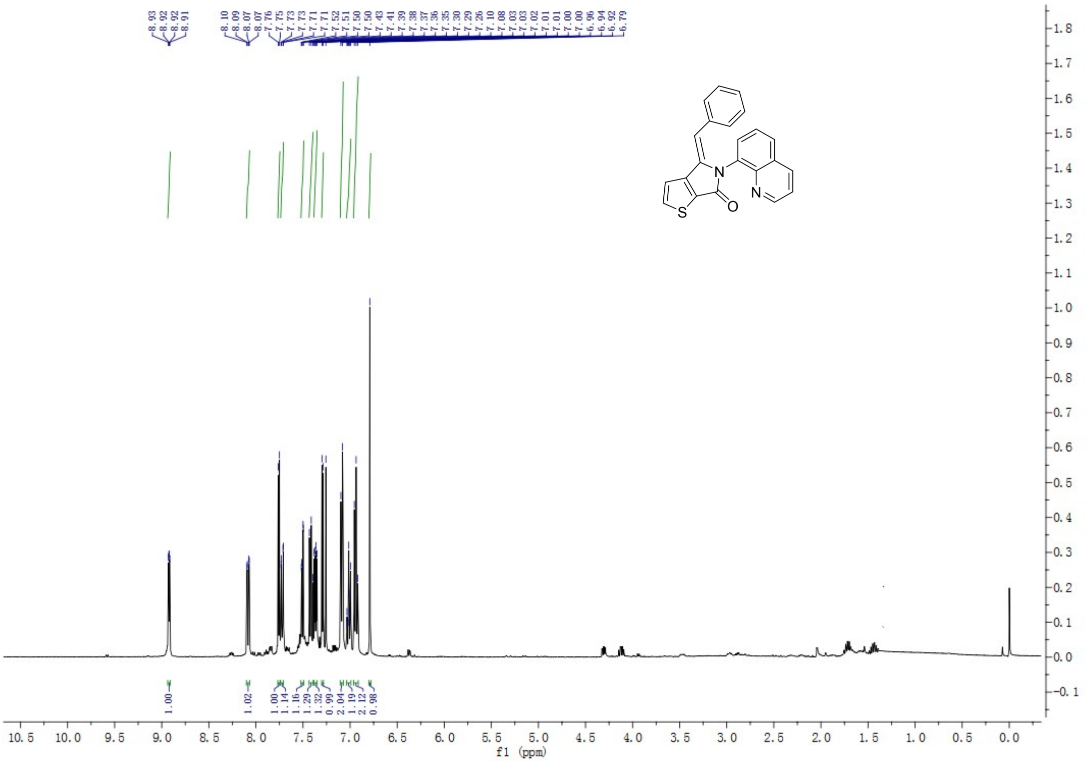

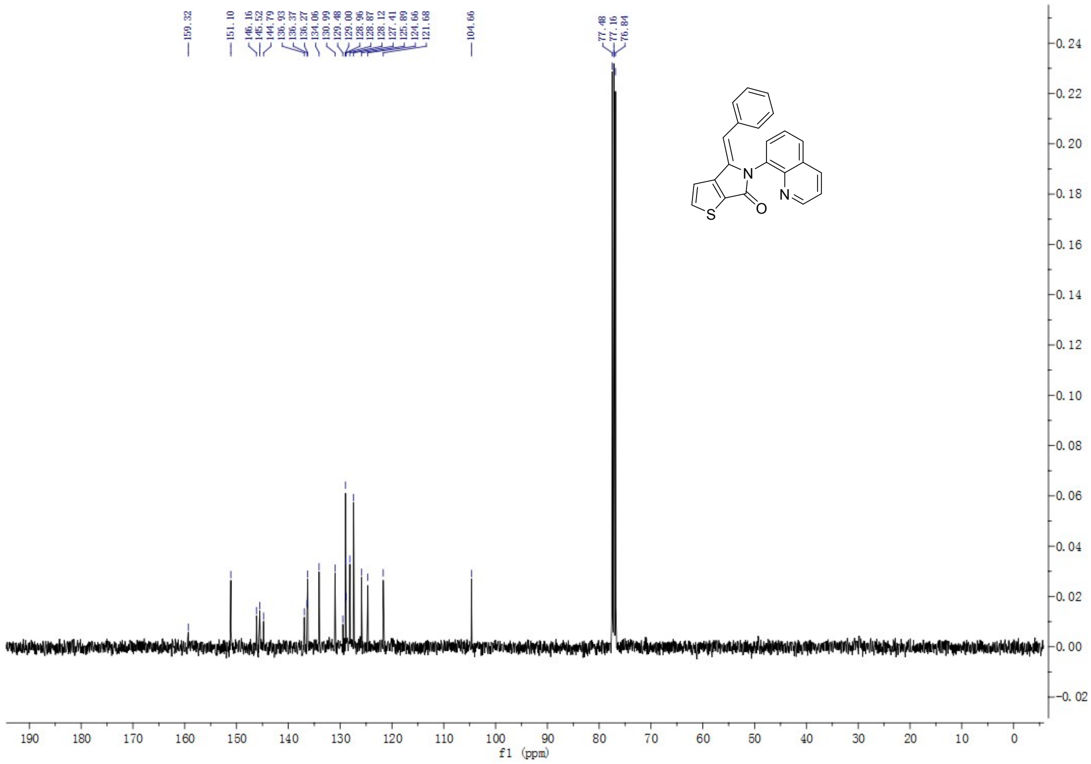

(*Z*)-3-(cyclohexylmethylene)-2-(quinolin-8-yl)isoindolin-1-one(3q)

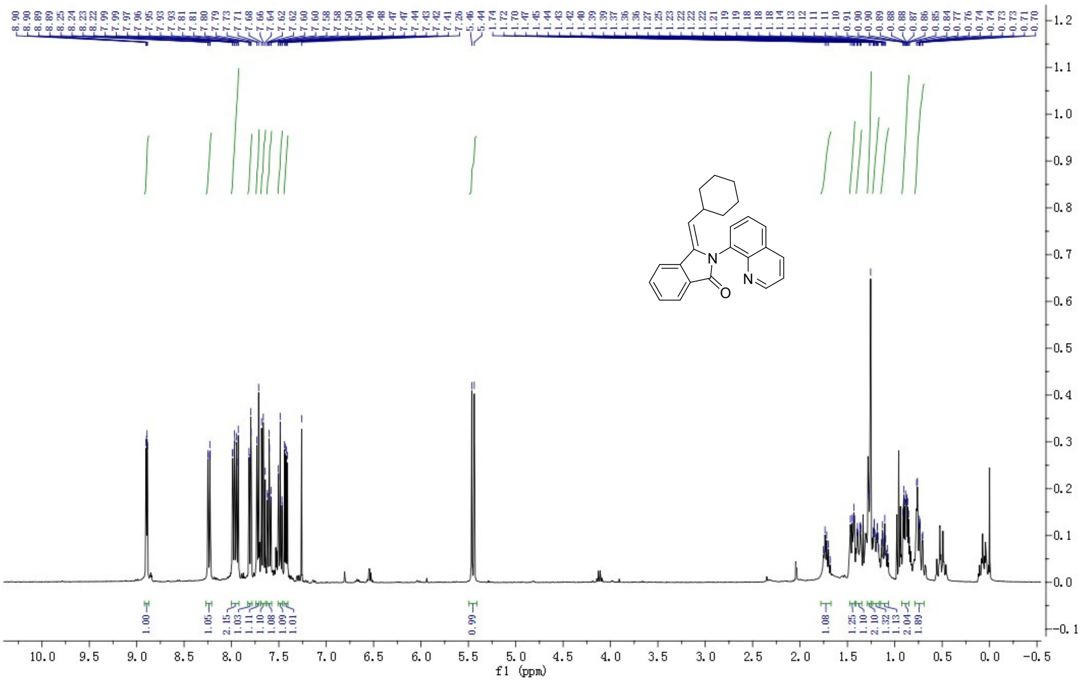

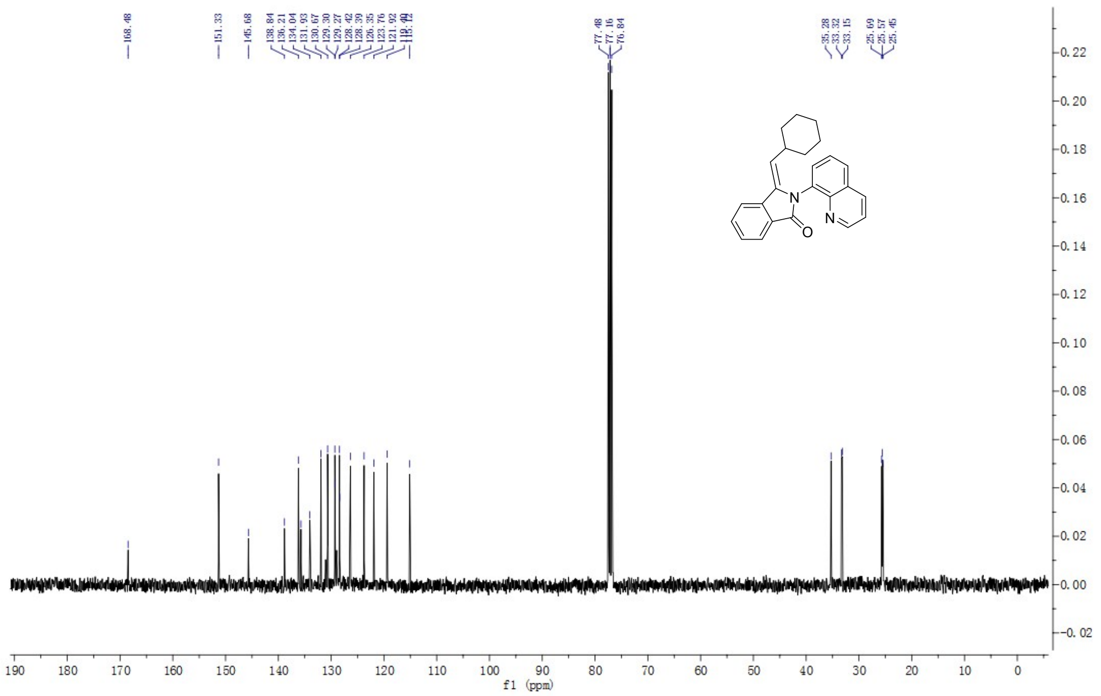

(*Z*)-3-(cyclopropylmethylene)-2-(quinolin-8-yl)isoindolin-1-one(3r)

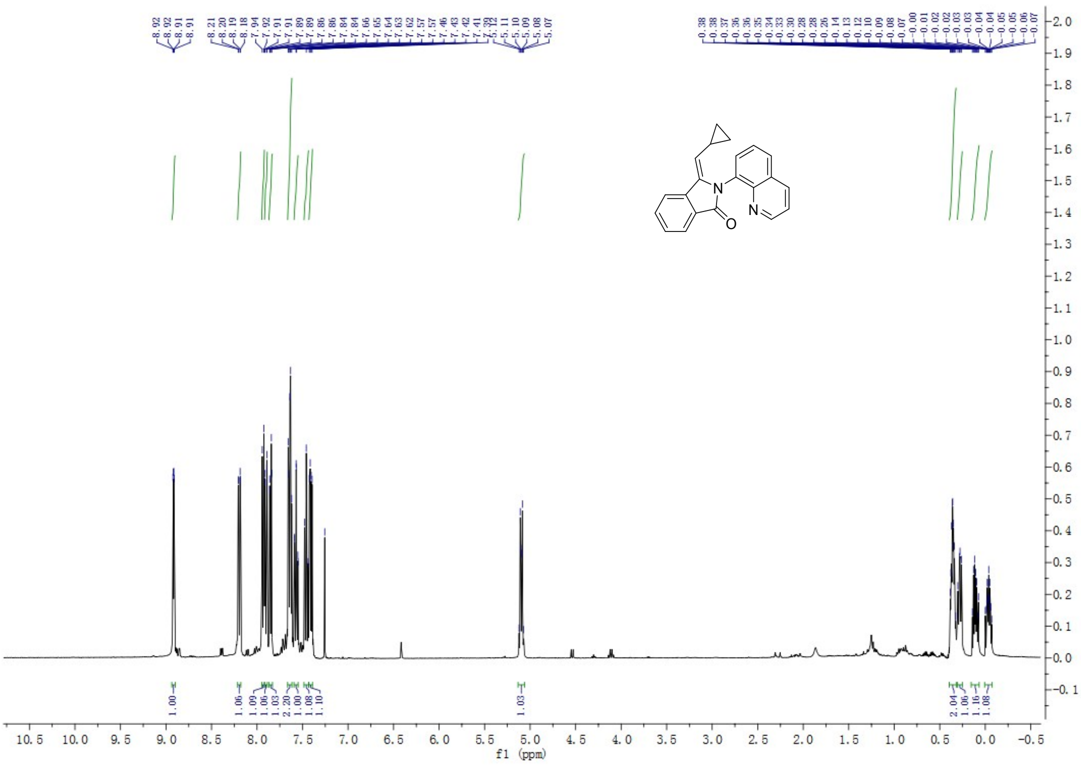

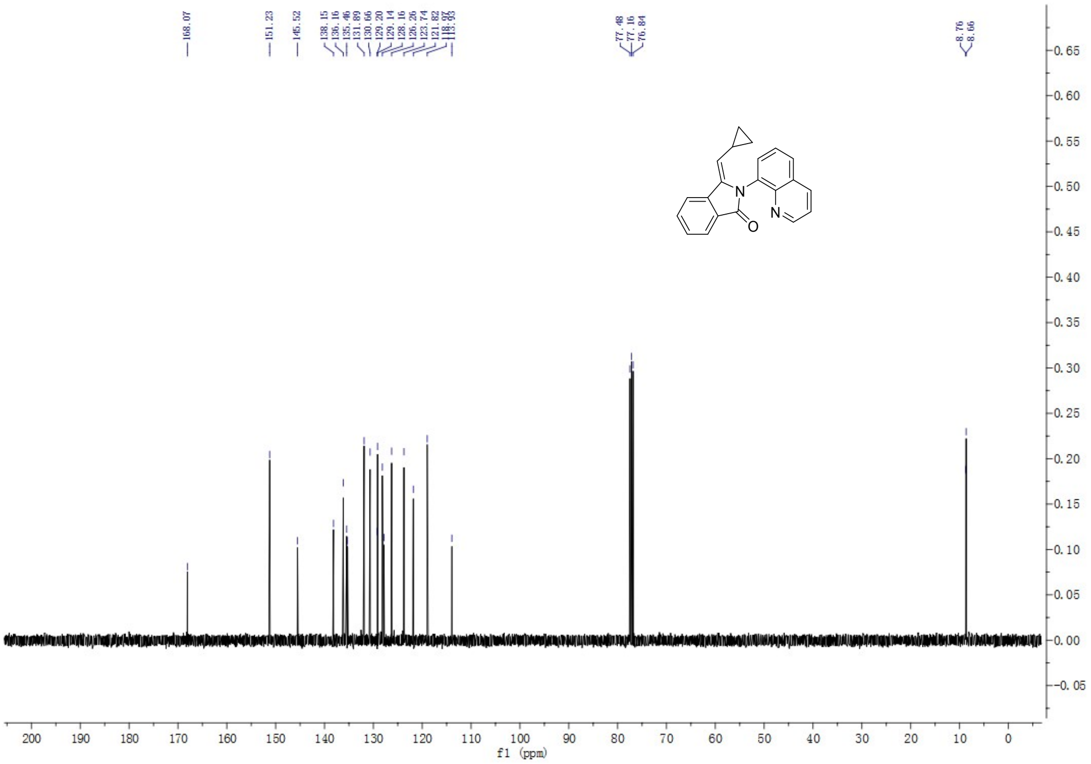

Supplement: RA-008-C8RA03278E-s001 [file RA-008-C8RA03278E-s001.pdf]
